# Prospective associations between psychosocial stress and the risk of type 2 diabetes in middle-aged adults: findings from the KoGES_CAVAS

**DOI:** 10.4178/epih.e2025061

**Published:** 2025-10-31

**Authors:** Ji Eun Kim, Hye Won Woo, Yu-Mi Kim, Min-Ho Shin, Sang Baek Koh, Mi Kyung Kim

**Affiliations:** 1 Department of Preventive Medicine, Hanyang University College of Medicine, Seoul, Korea; 2 Institute for Health and Society, Hanyang University, Seoul, Korea; 3 Department of Preventive Medicine, Chonnam National University Medical School, Gwangju, Korea; 4 Department of Preventive Medicine and Institute of Occupational Medicine, Yonsei University Wonju College of Medicine, Wonju, Korea

**Keywords:** Stress, Diabetes mellitus, Prospective studies, Metabolism, Insulin resistance, Abdominal obesity

## Abstract

**OBJECTIVES:**

Psychosocial stress is a potential risk factor for type 2 diabetes (T2D); however, the influence of the timing of stress exposure remains uncertain. We examined the prospective associations of baseline, cumulative average, and recent psychosocial stress with the risk of incident T2D in middle-aged adults.

**METHODS:**

We analyzed data from 7,880 participants aged 40-64 years without T2D at baseline. Psychosocial stress was assessed repeatedly using the Psychosocial Well-Being Index-Short Form. Incidence rate ratios (IRRs) and 95% confidence intervals (CIs) were estimated using modified Poisson regression models with robust error estimators.

**RESULTS:**

A total of 437 T2D cases occurred over 47,621 person-years. While baseline stress showed a non-significant association, both cumulative and recent stress demonstrated positive associations with T2D risk. Recent stress exhibited the strongest association in both male (stress vs. healthy group: IRR, 2.23; 95% CI, 1.41 to 3.52; highest [T3] vs. lowest tertile [T1]: IRR, 1.50; 95% CI, 1.07 to 2.10) and female (stress vs. healthy group: IRR, 1.72; 95% CI, 1.11 to 2.66; T3 vs. T1: IRR, 1.73; 95% CI, 1.26 to 2.37). These associations were more pronounced among participants with abdominal obesity, showing a significant positive linear trend (Bonferroni-corrected threshold, p=0.003).

**CONCLUSIONS:**

Recent psychosocial stress was associated with an increased incidence of T2D, underscoring the importance of integrating psychosocial factors into diabetes prevention strategies, particularly for individuals with abdominal obesity.

## GRAPHICAL ABSTRACT

[Fig f2-epih-47-e2025061]

## Key Message

Recent psychosocial stress was identified as the strongest risk factor for type 2 diabetes (T2D), showing non-significant relationship with baseline stress and weaker link for cumulative stress. Particularly, it was more pronounced among those with abdominal obesity, highlighting the importance of stress management in T2D prevention focusing on this high-risk subgroup.

## INTRODUCTION

Type 2 diabetes (T2D) is a major global health concern due to its steadily increasing prevalence in recent years [1]. According to the International Diabetes Federation, approximately 463 million individuals were living with diabetes worldwide in 2019, and this number is projected to rise to 700.2 million by 2045 [2]. A similar upward trend has been observed in Korea, where the prevalence of T2D increased from 7.9% in 2009 to 12.4% in 2021 [3]. Consequently, identifying modifiable risk factors is critical for developing effective public health strategies to prevent T2D onset. However, the incidence of T2D continues to rise despite efforts to address conventional risk factors such as obesity, sedentary behavior, and poor diet [4-7]. This trend highlights the need to identify additional contributing factors.

One such factor is psychosocial stress, which results from an imbalance between external stressors (e.g., job stress, unemployment, and social disadvantage) and coping mechanisms (e.g., personal resilience, social support, and psychological interventions) [8]. Psychosocial stress may promote T2D development through physiological pathways such as chronic activation of the hypothalamic-pituitary-adrenal (HPA) axis, increased cortisol secretion, and impaired glucose metabolism [9,10]. Several large-scale prospective cohort studies, including the Whitehall II study in the United Kingdom [5] and population-based cohorts in Sweden [11] and Australia [12], have reported that psychosocial or work-related stress is associated with an elevated T2D risk, particularly among female. Nevertheless, these associations have not been consistently observed. Some studies reported no significant correlations in female [13,14], suggesting that heterogeneity may arise from differences in population characteristics, sex, or methods of stress assessment [11-14]. Consequently, the relationship between psychosocial stress and T2D risk remains inconclusive, warranting further investigation.

Mechanistically, prolonged exposure to stress can trigger chronic physiological responses that disrupt glucose regulation [9]. Even short-term stress may induce peripheral insulin resistance and alter insulin sensitivity within an hour of peak stress exposure [15], potentially accelerating the pathogenesis of T2D [16,17]. However, most prior studies have relied on a single measure of psychosocial stress, which may not adequately capture its dynamic and fluctuating nature. Earlier research has primarily emphasized baseline assessments, overlooking both the long-term accumulation of stress and its short-term variations prior to T2D onset. This methodological limitation may partly account for the inconsistent findings in previous studies regarding stress–T2D associations.

In this study, we hypothesized that both cumulative and recent psychosocial stress would be associated with an increased risk of developing T2D and that these associations might differ according to the timing and duration of stress exposure. By differentiating between baseline, cumulative, and recent stress levels, we aimed to provide a more comprehensive understanding of the prospective relationship between psychosocial stress and incident T2D in middle-aged adults aged 40-64 years.

## MATERIALS AND METHODS

### Study design and population

The Korean Genome and Epidemiology Study–Cardiovascular Disease Association Study (KoGES_CAVAS) was established to comprehensively investigate risk factors associated with cardiometabolic health, including T2D [18]. The KoGES_CAVAS comprises 3 cohorts [18]: the Multi-Rural Communities Cohort (MRCohort), the Atherosclerosis Risk of Rural Areas in the Korean General Population (ARIRANG) cohort, and the Kanghwa cohort. Individuals aged ≥40 years were recruited from community settings through multistage cluster sampling.

A total of 19,546 participants without cardiovascular disease (CVD) or cancer (9,759 from the MRCohort, 5,942 from ARIRANG, and 3,845 from Kanghwa) were enrolled between 2005 and 2011 and followed up every 2-4 years between 2007 and 2017. The median follow-up duration was 5.96 years (interquartile range [IQR], 3.17–8.76), with 78.2% of participants attending more than 1 follow-up visit. Participants from the Kanghwa cohort (n=3,845) were excluded because of missing data for the exposure variable—the Psychosocial Well-Being Index–Short Form (PWI-SF), which measures psychosocial stress. As PWI-SF data were collected only for adults younger than 65 years, participants aged ≥65 years were excluded (n=5,097). Those who reported using anti-diabetic drugs or insulin or had fasting blood glucose (FBG) levels ≥126 mg/dL (7.0 mmol/L) at baseline were also excluded (n=1,090). Participants with missing data for PWI-SF scores (n=1,288) or for key covariates such as education level, regular exercise, smoking status, alcohol consumption, body mass index (BMI), or Diet Quality Index–International (DQI-I) (n=346) were further excluded. The final analytic sample consisted of 7,880 participants (2,878 male and 5,002 female) (Supplementary Material 1).

### Assessment of psychosocial stress

Psychosocial stress was evaluated using the PWI-SF, derived from the General Health Questionnaire [19], which has been validated as a reliable measure in the Korean population [20]. The PWI-SF demonstrated high internal consistency (Cronbach’s α=0.90) and excellent convergent validity with Goldberg’s General Health Questionnaire, a well-established measure of psychological well-being (r=0.98). The instrument includes 4 domains: (1) social performance and self-confidence (8 items; maximum score, 24), (2) depression (3 items; maximum score, 9), (3) sleep disturbance and anxiety (3 items; maximum score, 9), and (4) overall well-being and vitality (4 items; maximum score, 12). Each item is rated on a 4-point Likert scale (“not at all,” “sometimes,” “often,” and “always”), yielding total scores from 0 to 54, with higher scores indicating greater psychosocial stress. The PWI-SF was self-administered. Participants were categorized using 2 classification schemes based on stress scores: (1) predefined cutoff values–healthy group (score ≤8), potential stress group (score >8 and <27), and stress group (score ≥27) [20]; (2) tertile-based classification–scores divided into tertiles to form 3 equally sized groups for analysis.

These classifications were applied to 3 distinct measures of psychosocial stress: baseline, cumulative average, and recent scores. Baseline PWI-SF scores captured stress levels reflecting a relatively long latency period. Cumulative average scores represented long-term aggregated stress exposure across multiple visits. Recent PWI-SF scores were defined as the most recent measurement prior to T2D diagnosis for incident cases or prior to the end of follow-up for non-cases.

### Ascertainment of diabetes incidence

At baseline and each follow-up visit, participants reported whether they had been diagnosed with T2D by a physician and whether they were receiving anti-diabetic drug or insulin therapy. Incident T2D cases were defined as participants who met either of the following criteria: (1) a new physician diagnosis of T2D followed by prescription of oral medication or insulin; or (2) an FBG level ≥126 mg/dL (7.0 mmol/L) at the follow-up visit.

Person-years of follow-up were calculated from the date of enrollment until the earliest of the following events: T2D diagnosis, diagnosis of CVD or cancer (to minimize confounding by subsequent treatments), or the end of follow-up. For participants lost to follow-up, follow-up time was assigned as half the median duration of participants who completed the corresponding visit interval, assuming uniform censoring within that period [21,22]. For participants who reported a diagnosis of CVD or cancer but lacked an exact date, the diagnosis time was imputed as the midpoint between the last completed and next reported visits [21,22].

### Assessment of covariates

Covariate data were obtained through face-to-face interviews and clinical examinations conducted by trained interviewers and examiners using standardized protocols. Demographic characteristics (e.g., age, sex, and education level) and lifestyle behaviors related to T2D risk (regular exercise, alcohol consumption, smoking status, and DQI-I) were collected. Height and weight were measured to the nearest 0.1 cm and 0.1 kg, respectively, with participants wearing light clothing and no shoes. BMI was calculated as weight (kg) divided by height squared (m²). Venous blood samples were collected after an 8-hour overnight fast, and serum FBG concentrations were measured using an ADVIA 1650 Chemistry Analyzer (Siemens, Tarrytown, NY, USA).

### Statistical analysis

Statistical analyses were performed separately for male and female. Descriptive statistics are presented as the mean and standard deviation (SD) for continuous variables and as numbers (n) and percentages (%) for categorical variables. A general linear model was used to calculate age-adjusted means and standard errors (SEs) or percentages (%) for general characteristics. Linear trends were assessed by treating the median value of each category or tertile as a continuous variable. Unadjusted Kaplan–Meier survival curves were generated by categories of recent PWI-SF scores for male and female, and log-rank tests were applied to compare survival distributions across categories.

Incidence rate ratios (IRRs) and corresponding 95% confidence intervals (CIs) were estimated using modified Poisson regression models with a log link and robust error variance. Person-time was included as an offset term (log of person-years) to account for varying follow-up durations. Although Cox proportional hazards models are typically used for time-to-event analyses, modified Poisson regression was selected in this study for 2 main reasons: (1) incident T2D was primarily ascertained at discrete health examination visits rather than through continuous observation, and (2) participants contributed differing follow-up lengths, necessitating a person-time–based approach [23]. Moreover, the modified Poisson method has been recommended for providing direct and robust estimation of IRRs [24]. The “healthy group” or the lowest tertile (T1) served as the reference group. We considered 3 models: (1) an age-adjusted model; (2) a directed acyclic graph (DAG)-informed model, adjusted for minimal adjustment set identified in dagitty version 3.1 (https://dagitty.net/mWXJJFWxx; Supplementary Material 2), which included age (years) and educational attainment (≥12 years of education, yes or no); and (3) a fully adjusted model that included additional covariates, including regular exercise (≥3 times/wk and ≥30 min/session, yes or no), smoking status (never, past, or current in male and current smoking status, yes or no in female), alcohol consumption (mL/day), DQI-I, and BMI. These covariates were selected based on previous evidence linking psychosocial stress to metabolic disorders and an increased risk of T2D [5,9,12], partly through adverse behavioral changes, such as an unhealthy diet, physical inactivity, smoking, and excessive alcohol consumption [25], all of which can contribute to obesity, insulin resistance, and T2D [26-28]. To examine potential interactions between psychosocial stress (PWI-SF scores) and T2D risk factors, stratified analyses were conducted by education level (<12 or ≥12 years), regular exercise (yes or no), smoking and drinking status (never/past or current), BMI (<23 or ≥23 kg/m²), and prediabetes status (FBG <100 or 100-125 mg/dL at baseline). Interactions were evaluated using cross-product terms in Poisson regression models, and within each stratum, median PWI-SF values were used to estimate p-trend values.

To further assess dose-response relationships between psychosocial stress and T2D risk, 2 additional analyses were performed. First, restricted cubic spline models with 3 knots placed at the 25th percentile, 50th percentile, and 75th percentile of the PWI-SF distribution were used to model continuous associations and to test for non-linearity. Second, to provide a more clinically interpretable risk estimate, the PWI-SF score was analyzed as a continuous variable, and IRRs with 95% CIs were calculated per 5-point increase in score within the multivariable model.

Sensitivity analyses were conducted to evaluate the robustness of the findings. To address potential biases in outcome ascertainment, participants diagnosed with CVD or cancer between visits were censored to minimize treatment-related effects on T2D incidence, T2D events occurring within the first year were excluded to reduce reverse causation bias, and analyses were restricted to participants with at least 2 follow-up visits. To account for socio-demographic influences, analyses were repeated among married participants only, given the known relationships between marital status, stress, and T2D risk [29] and the high proportion of married individuals in this cohort (male, 89.7%; female, 83.9%). To address potential clinical and methodological confounding, additional models were adjusted for baseline FBG as a continuous variable, reanalyzed after excluding participants with prediabetes at baseline, included cohort (MRCohort and ARIRANG) as a fixed effect to account for potential clustering by recruitment site, applied a stricter definition of T2D based solely on medication treatment, and repeated the analyses after excluding BMI from the model. All analyses were performed using SAS version 9.4 (SAS Institute Inc., Cary, NC, USA).

The study was reported following the Strengthening the Reporting of Observational Studies in Epidemiology (STROBE) guidelines, and the completed checklist is provided in Supplementary Material 3.

### Ethics statement

This study was conducted in accordance with the principles of the Declaration of Helsinki, and the protocol was approved by the Institutional Review Board of Hanyang University (HYUIRB-202403-025). All participants provided written informed consent prior to study enrollment.

## RESULTS

### General characteristics at baseline

The general characteristics of participants are summarized in Supplementary Material 4. Among the 7,880 participants (male, 36.5%; female, 63.5%) contributing a total of 47,621 person-years, 437 developed T2D (male, 203; female, 234). The mean ages of male and female were 53.8 years and 52.7 years, respectively. Compared with female, male were more likely to have higher educational attainment, to be current smokers or drinkers, and were less likely to engage in regular exercise. Male also exhibited larger waist circumference (WC) and higher FBG levels, but lower PWI-SF scores, indicating lower psychosocial stress. The median follow-up duration was 4.57 years (IQR, 2.36-7.53) for incident T2D cases and 6.42 years (IQR, 3.53-8.86) for non-cases. The median lag time between the most recent PWI-SF assessment and T2D diagnosis among incident cases was 33.3 months (IQR, 22.1-47.9), with a median of 2 visits (IQR, 1.0-3.0). In the comparison of age-adjusted and sex-adjusted baseline characteristics between the final study population and excluded participants (Supplementary Material 5), those excluded were significantly older, less educated, less physically active, and more likely to be current smokers. Higher recent PWI-SF scores were associated with lower T2D-free survival, as shown in Supplementary Material 6. In male, group differences were significant in both categorical stress groups (log-rank p=0.004) and tertiles (p=0.027). In female, the differences were significant across tertiles (p=0.008) and borderline for categorical groups (p=0.059).

Table 1 presents age-adjusted baseline characteristics stratified by baseline psychosocial stress. Among male, those in the high-risk and highest tertile (T3) groups tended to be younger, more likely to exercise and smoke, and had lower BMI, WC, and FBG levels, as well as lower education levels. Female demonstrated similar trends, although differences in age, BMI, and WC were not statistically significant. The proportion of menopausal female increased with stress level, while drinking status showed no clear linear trend in either sex. The characteristics of cumulative and recent stress are presented in Supplementary Material 7, which showed similar patterns: higher-stress groups were younger, more likely to smoke, and had lower BMI, WC, and FBG levels. Educational attainment also varied by stress classification.

### Association of psychosocial stress with the risk of type 2 diabetes

In both male and female, cumulative and recent psychosocial stress were significantly associated with T2D risk, whereas baseline stress was not (Table 2). Crude T2D incidence rates increased with higher recent psychosocial stress, ranging from 9.3 to 20.0 per 1,000 person-years in male and from 6.1 to 10.0 in female. Similar, though less pronounced, gradients were observed for cumulative stress, whereas baseline stress showed little variation across categories. After adjustment for age and education (multivariable model 1, derived from a DAG), a strong positive association was observed between recent psychosocial stress and T2D risk. These associations remained significant after further adjustment for lifestyle factors and BMI (multivariable model 2, fully adjusted). In the fully adjusted model, the IRRs for male with recent stress were 2.23 (95% CI, 1.41 to 3.52; p-trend=0.002) for the high-risk group (vs. healthy group) and 1.50 (95% CI, 1.07 to 2.10; p-trend=0.018) for the highest tertile (T3 vs. T1). Similarly, female had IRRs of 1.72 (95% CI, 1.11 to 2.66; p-trend=0.008) for the “high-risk group” and 1.73 (95% CI, 1.26 to 2.37; p-trend=0.001) for T3. Cumulative average stress scores also showed positive associations. Among male, the high-risk group demonstrated an IRR of 2.07 (95% CI, 1.23 to 3.47; p-trend=0.035). Among female, a significant association was observed in the highest tertile (IRR, 1.50; 95% CI, 1.07 to 2.10; p-trend=0.018). No significant interaction was found between sex and psychosocial stress categories (all interaction p-values>0.05).

**Figure 1.**
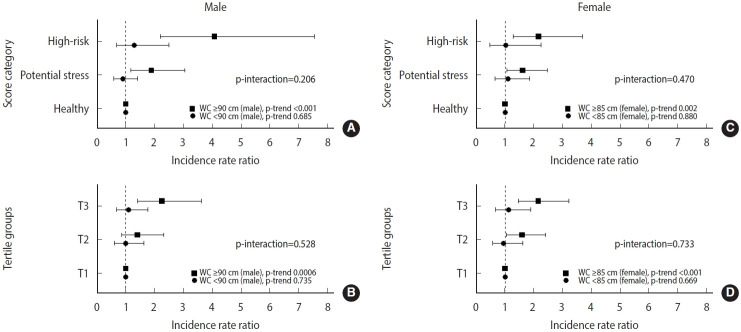
Stratified association between recent psychosocial stress and the incidence of type 2 diabetes by waist circumference (WC) in male and female. (A) and (B) present the results for male, and (C) and (D) present the results for female. Psychosocial stress was categorized using either score-based categories (healthy, potential stress, high risk; (A) and (C)) or tertile groups (T1-T3; [B] and [D]). Analyses were stratified by WC: WC ≥90 cm vs. <90 cm for male and WC ≥85 cm vs. <85 cm for female. Multivariable Poisson regression models were adjusted for age, education (≥12 years), regular exercise (≥3 times/wk, ≥30 min/session), smoking (current, past, or never for male; yes or no for female), alcohol intake (mL/day), body mass index (kg/m²), and Diet Quality Index-International. The p-trend values indicate linear trends across stress categories within each WC group.

Stratified analyses were conducted for 7 covariates (Supplementary Materials 8 and 9). None of the interactions reached the Bonferroni-corrected threshold for significance (p<0.007, derived as 0.05/7). However, a significant positive linear trend was observed exclusively among participants with abdominal obesity, as defined by WC, at a Bonferroni-corrected threshold of 0.0035 (0.05/14) across both recent psychosocial stress classification schemes in male (Figure 1A and B) and female (Figure 1C and D).

When PWI-SF scores were treated as continuous variables, the results supported a dose–response relationship (Table 2). A 5-point increase in the recent PWI-SF score was significantly associated with higher T2D risk in both male (IRR, 1.13; 95% CI, 1.04 to 1.22) and female (IRR, 1.09; 95% CI, 1.03 to 1.16). Analyses using restricted cubic splines visually confirmed this positive trend, with no significant evidence of non-linearity (p for non-linearity >0.05 for all models), as illustrated in Supplementary Material 10. The dose–response curve was particularly steep for participants with abdominal obesity.

### Sensitivity analyses

Table 3 presents the associations under multiple conditions: (1) inclusion of participants with CVD or cancer, (2) exclusion of cases occurring within the first follow-up year, and (3) exclusion of those who did not participate in any follow-up surveys. Additionally, (4) analyses restricted to married participants yielded consistent results. Across all scenarios, the associations, particularly for recent and cumulative average PWI-SF scores, remained robust. Furthermore, the associations persisted after (5) including baseline FBG as a covariate and (6) excluding participants with prediabetes at baseline (Table 3), confirming robustness against potential confounding by initial glycemic status. Although the interaction between psychosocial stress and baseline glycemic category (normoglycemia vs. prediabetes) was not statistically significant, the associations appeared slightly stronger among participants with normoglycemia (Supplementary Materials 8 and 9). Similarly, (7) after accounting for cohort clustering and (8) after excluding BMI from the main multivariable model, the results showed only minimal numerical differences, and statistical significance was maintained. Finally, (9) using a stricter definition of T2D diagnosis (restricted to medication-only cases representing more severe disease) produced results consistent with the primary findings, confirming that conclusions were not driven by inclusion of FBG-defined cases alone (Table 3).

## DISCUSSION

In this prospective cohort study of middle-aged adults, we found that recent psychosocial stress was most strongly associated with an increased risk of T2D, while cumulative stress also showed a positive association and baseline stress did not. These findings suggest that the timing of stress exposure may be more critical than the overall accumulated burden. Notably, the association between recent stress and T2D was more pronounced among participants with abdominal obesity. The robustness of these associations across multiple sensitivity analyses further supports their reliability.

In this study, T2D risk exhibited a stronger relationship with recent stress compared with baseline or cumulative stress. The absence of a clear association between baseline stress and T2D may reflect the inability of a single measurement taken several years before diagnosis to adequately capture subsequent changes in stress exposure [12,30]. The modest association observed with cumulative stress might be due to the averaging approach, which can dilute the influence of short-term, high-intensity stress episodes. Indeed, acute stress has been shown to trigger immediate physiological responses, such as inflammatory activation and dysregulation of the HPA axis [31], which may more directly contribute to the onset of T2D. Supporting this, a 12-year longitudinal study reported that moderate or high stress assessed 3 years earlier was associated with more than a twofold increase in diabetes risk, with much of this effect unexplained by traditional risk factors [30]. Collectively, these findings indicate that the temporal proximity of stress assessment may be more influential than the total cumulative burden in predicting T2D development.

Mechanistically, psychosocial stress measured by the PWI-SF reflects stress experienced over the preceding few weeks, thereby capturing transient or acute stress rather than long-term chronic stress. Many prior studies have relied on single-point measures of perceived stress or general mental health [12], which may have limited their capacity to detect the metabolic consequences of short-term stress. Evidence from human and animal studies on acute stress and metabolic health provides valuable insight into the underlying biological mechanisms [32,33]. Experimental research has shown that acute stress can temporarily elevate blood glucose through increased secretion of cortisol and catecholamines [31], while animal models demonstrate that repeated acute stress leads to persistent HPA axis activation, thereby disrupting glucose homeostasis over time [34]. Recurrent activation of stress pathways may impair glucose metabolism and increase the risk of T2D [35]. Moreover, adrenaline and noradrenaline, which are released during acute stress, can transiently increase hepatic glucose output and reduce insulin sensitivity as part of the “fight-or-flight” response [31]. Although isolated stress episodes may be adaptive, frequent or intense exposures can contribute to lasting metabolic dysregulation. Accordingly, stress assessed in close temporal proximity to disease onset may better capture these acute, direct effects, whereas single or averaged measurements collected years earlier may underestimate the true impact of stress on T2D risk. Although there was no statistically significant interaction by baseline glycemic status, the slightly stronger association observed among participants with normoglycemia at baseline warrants attention. Several mechanisms may explain this pattern. First, individuals with prediabetes already possess elevated baseline risk, which could attenuate the relative effect of psychosocial stress, making the association more evident among normoglycemic participants [11]. Second, if stress contributed to prediabetes development prior to baseline, the subsequent stress–disease pathway may have already been activated, thereby diminishing observable effects [36]. Finally, reverse causation or reporting bias is less likely in normoglycemic individuals, which may allow for a more accurate estimation of the prospective stress–T2D relationship [11].

The inconsistencies in earlier findings may partly be attributable to sex differences across study populations. Some studies have suggested that the metabolic effects of stress differ by sex owing to hormonal and behavioral factors [1,16,37]. Furthermore, stratified analyses of covariates in this study revealed no significant effect modifications or interactions, except for WC, which showed a positive association. Given that abdominal obesity is closely linked to insulin resistance, individuals with higher WC may be more vulnerable to stress-related metabolic dysfunction [38]. Central (visceral) obesity is more metabolically active than general adiposity and contributes to insulin resistance through the secretion of pro-inflammatory cytokines and the dysregulation of cortisol metabolism [1,9]. This finding is consistent with previous evidence showing that central adiposity mediates the relationship between psychosocial stress and metabolic disturbances. Prolonged exposure to stress-related hormones such as cortisol may promote visceral fat accumulation, further exacerbating insulin resistance [1,16]. Additional research is needed to clarify these pathways and to develop targeted interventions for high-risk subgroups, such as individuals with abdominal obesity. In this regard, our findings have practical implications for T2D prevention, as the strong association with recent stress underscores the importance of timely intervention. For instance, routine stress assessments could be integrated into national health screening programs, and community-based initiatives, such as stress management workshops, counseling, or lifestyle modification programs, could be implemented, particularly for high-risk populations such as female with abdominal obesity.

Despite rigorous methodological considerations, several limitations should be acknowledged. First, the dataset exhibited a relatively low complete follow-up rate and uneven follow-up durations across participants, which may have introduced bias. Second, approximately 17.6% of participants had missing data, primarily owing to incomplete self-administered PWI-SF responses (13.5%), although missingness for outcomes or other covariates was relatively low (4.2%). To address this, we conducted multiple imputation analyses, which yielded results consistent with those from the complete-case analysis. In the fully adjusted model, the IRR for recent stress was 2.26 (95% CI, 1.45 to 3.53) in male and 1.67 (95% CI, 1.22 to 2.27) in female, closely matching the complete-case estimates (Supplementary Material 11). Third, covariates were selected based on prior evidence from meta-analyses and cohort studies emphasizing factors known to be associated with psychosocial stress and T2D risk. Nonetheless, the possibility of unmeasured or residual confounding cannot be excluded. For example, we used only baseline values for variables such as smoking, alcohol consumption, and physical activity, without accounting for potential changes during follow-up. Additionally, several potential confounders, including genetic predisposition, income level, and comorbid mental health conditions [29], were unavailable. As a result, both time-varying and unmeasured confounding remain possible. To further evaluate this, we calculated E-values for the main associations. For the high-risk group and highest tertile of recent PWI-SF scores, the E-values were 3.88 and 2.37 in male and 2.86 and 2.86 in female, respectively, suggesting that relatively strong unmeasured confounders would be required to fully account for these associations. Thus, our findings appear moderately robust to potential unmeasured confounding [39]. Fourth, because most validation of the PWI-SF has been conducted in worker populations, it remains uncertain whether the instrument performs equivalently in general community samples, which may limit the generalizability of our findings. Fifth, the diagnostic criteria used in this study did not include hemoglobin A1c or the oral glucose tolerance test, potentially limiting comparability with studies employing those measures. Finally, as mortality data were not linked in this cohort, we could not formally account for death as a competing risk. However, given the middle-aged population (40-64 years) and moderate follow-up period, the potential influence of mortality on our results is likely minimal.

Despite these limitations, this study possesses several notable strengths. Its prospective cohort design enabled temporal assessment of psychosocial stress preceding T2D onset, reducing recall bias. Trained personnel collected data using standardized protocols, ensuring high data quality. To our knowledge, this is the first study to investigate the association between T2D risk and periodic psychosocial stress measures in a large Korean cohort.

In conclusion, this prospective cohort study of middle-aged adults demonstrat- ed that recent psychosocial stress was strongly associated with an increased risk of type 2 diabetes, whereas cumulative stress showed a weaker positive association. The association with recent stress was particularly pronounced among individuals with abdominal obesity, underscoring the importance of the timing of exposure in stress-related metabolic risk. Collectively, these findings highlight the need to consider psychosocial stress as an important risk factor in both clinical and public health approaches to diabetes prevention and risk stratification in community populations.

## Figures and Tables

**Figure f2-epih-47-e2025061:**
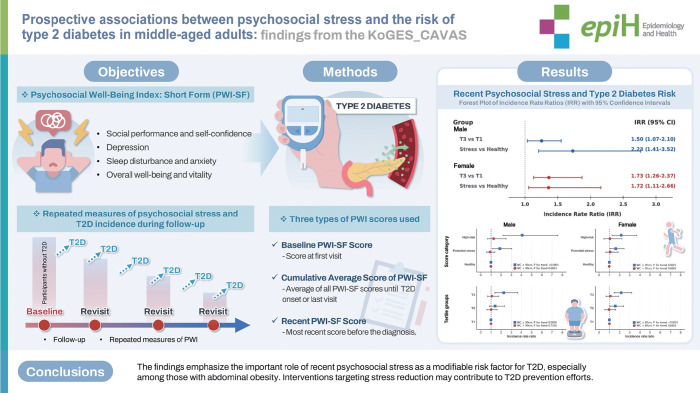


**Table 1.** Age-adjusted baseline characteristics of study participants according to psychosocial stress levels measured using the PWI-SF

Characteristics	Categories of baseline PWI-SF scores			p-trend^1^	Tertiles of baseline PWI-SF scores			p-trend^2^
	Healthy group (PWI-SF≤8.0)	Potential stress group (8.0<PWI-SF<27.0)	High-risk group (PWI-SF≥27.0)		T1	T2	T3	
Male								
n (%)	591 (20.5)	1,959 (68.1)	328 (11.4)		915 (31.8)	957 (33.3)	1,006 (35.0)	-
Median (Min-Max)^2^	4.0 (0.0-8.0)	16.0 (9.0-26.0)	30.0 (27.0-48.0)		7.0 (0.0-11.0)	15.0 (12.0-18.0)	24.0 (19.0-48.0)	-
Age (yr)	55.0±0.3^a^	53.4±0.1^b^	53.9±0.4^b^	0.001	54.6±0.2^a^	53.2±0.2^b^	53.6±0.2^b^	0.004
Higher education^3^	46.7^a^	48.3^a^	34.9^b^	0.003	48.9^a^	51.6^a^	39.2^b^	<0.001
Regular exercise^4^	28.1^a^	20.3^b^	11.6^c^	<0.001	26.3^a^	20.7^b^	16.3^c^	<0.001
Current smoker	34.5^b^	38.1^b^	50.4^a^	<0.001	35.1^b^	37.0^b^	43.8^a^	<0.001
Current drinker	68.2	72.3	67.7	0.787	69.1	72.8	70.8	0.467
Alcohol consumption (g/day)	23.9±1.7	24.3±0.9	25.0±2.3	0.702	24.5±1.4	23.6±1.3	24.8±1.3	0.841
Body mass index (kg/m^2^)	24.6±0.1^a^	24.6±0.1^a^	24.0±0.2^b^	0.003	24.7±0.1^a^	24.6±0.1^a^	24.2±0.1^b^	<0.001
Waist circumference (cm)	86.3±0.3^a^	86.5±0.2^a^	84.9±0.4^b^	0.027	86.7±0.3^a^	86.5±0.3^a^	85.7±0.2^b^	0.002
Fasting blood glucose (mg/dL)	96.3±0.4^a^	96.4±0.2^a^	94.6±0.6^b^	0.037	96.5±0.3^a,b^	96.6±0.3^a^	95.5±0.3^b^	0.024
Female								
n (%)	781 (15.6)	3,324 (66.5)	897 (17.9)		1,609 (32.2)	1,678 (33.6)	1,715 (34.3)	-
Median (Min-Max)^2^	5.0 (0.0-8.0)	17.0 (9.0-26.0)	31.0 (27.0-54.0)		9.0 (0.0-13.0)	17.0 (14.0-21.0)	27.0 (22.0-54.0)	-
Age (yr)	53.5±0.2^a^	52.3±0.1^b^	53.3±0.2^a^	0.888	52.9±0.2^a^	52.2±0.2^b^	52.9±0.2^a^	0.680
Higher education^3^	36.4^a^	35.0^a^	19.7^b^	<0.001	37.4^a^	36.7^a^	23.7^b^	<0.001
Regular exercise^4^	36.3^a^	25.3^b^	16.3^c^	<0.001	34.3^a^	24.9^b^	17.6^c^	<0.001
Current smoker	1.3^b^	1.7^b^	3.0^a^	0.005	1.4	1.7	2.4	0.035
Current drinker	33.7^a,b^	29.5^b^	34.7^a^	0.384	31.4^a,b^	28.9^b^	32.9^a^	0.247
Alcohol consumption (g/day)	2.3±0.4^a,b^	2.0±0.2^b^	3.0±0.3^a^	0.102	2.2±0.3	2.1±0.2	2.4±0.2	0.483
Body mass index (kg/m^2^)	24.8±0.1^a^	24.5±0.1^b^	24.7±0.1^a,b^	0.765	24.7±0.1	24.4±0.1	24.6±0.1	0.496
Waist circumference (cm)	82.7±0.3^a^	81.4±0.1^b^	82.1±0.3^a,b^	0.344	82.3±0.2^a^	81.2±0.2^b^	81.8±0.2^a,b^	0.123
Fasting blood glucose (mg/dL)	92.8±0.3^a^	92.0±0.2^b^	91.7±0.3^b^	0.015	92.6±0.2^a^	91.9±0.2^b^	91.8±0.2^b^	0.011
Menopausal status	62.8^b^	65.3^a,b^	67.2^a^	0.009	63.7^b^	65.2^a,b^	66.7^a^	0.010

All values were adjusted for age (otherwise specified) and expressed as mean±standard error for continuous variables or percentages for categorical variables; Mean values with different superscripts (a, b, c) within a row are significantly different between groups, according to Tukey’s multiple comparison test. PWI-SF, Psychosocial Well-Being Index-Short Form; Min, minimum; Max, maximum.

1 Linear trends were obtained by treating the median value of each group as a continuous variable.

2 PWI-SF score observed within each stress-level group or tertile.

3 Higher education level (≥12 years of education).

4 Regular exercise (≥3 times/wk and ≥30 min/session).

**Table 2.** Incidence rate ratios of type 2 diabetes according to score categories and tertile groups of psychosocial stress level using baseline, cumulative average, and recent PWI-SF scores

Variables	Categories of PWI-SF scores			p-trend^1^	Tertiles of PWI-SF scores			p-trend^1^	Per 5-point increase
	Healthy group (PWI-SF≤8.0)	Potential stress group (8.0<PWI-SF<27.0)	High-risk group (PWI-SF≥27.0)		T1	T2	T3		
Male									
Baseline									
n (%)	591 (20.5)	1,959 (68.1)	328 (11.4)		915 (31.8)	957 (33.3)	1,006 (35.0)		
Median (Min-Max)^2^	4.0 (0.0-8.0)	16.0 (9.0-26.0)	30.0 (27.0-48.0)		7.0 (0.0-11.0)	15.0 (12.0-18.0)	24.0 (19.0-48.0)		
No. of cases/person-years	40/3,377	136/11,960	27/1,976		64/5,260	81/5,902	58/6,151		
Age-adjusted model	1.00 (reference)	1.00 (0.70, 1.41)	1.19 (0.73, 1.93)	0.541	1.00 (reference)	1.17 (0.85, 1.62)	0.79 (0.56, 1.12)	0.146	1.00 (0.93, 1.09)
Multivariable model 1^3^	1.00 (reference)	0.99 (0.70, 1.41)	1.19 (0.73, 1.94)	0.538	1.00 (reference)	1.17 (0.85, 1.62)	0.79 (0.55, 1.13)	0.150	1.00 (0.93, 1.09)
Multivariable model 2^3^	1.00 (reference)	0.95 (0.67, 1.36)	1.15 (0.70, 1.90)	0.648	1.00 (reference)	1.15 (0.83, 1.60)	0.78 (0.54, 1.12)	0.145	1.00 (0.92, 1.09)
Cumulative average									
n (%)	593 (20.6)	2,073 (72.0)	212 (7.4)		995 (34.6)	937 (32.6)	946 (32.9)		
Median (Min-Max)^2^	5.0 (0.0-8.0)	15.0 (8.3-26.7)	30.0 (27.0-47.0)		7.3 (0.0-11.0)	14.0 (11.3-17.0)	22.0 (17.3-47.0)		
No. of cases/person-years	40/3,393	140/12,889	23/1,031		67/5,912	69/5,981	67/5,420		
Age-adjusted model	1.00 (reference)	0.96 (0.68, 1.36)	1.97 (1.19, 3.28)	0.086	1.00 (reference)	1.05 (0.76, 1.47)	1.12 (0.80, 1.56)	0.515	1.08 (0.99, 1.19)
Multivariable model 1^3^	1.00 (reference)	0.96 (0.68, 1.36)	1.98 (1.19, 3.30)	0.046	1.00(reference)	1.05 (0.76, 1.47)	1.12 (0.80, 1.56)	0.511	1.08 (0.99, 1.19)
Multivariable model 2^3^	1.00 (reference)	0.97 (0.68, 1.38)	2.07 (1.23, 3.47)	0.035	1.00 (reference)	1.02 (0.73, 1.44)	1.12 (0.79, 1.58)	0.510	1.09 (0.99, 1.20)
Recent									
n (%)	888 (30.9)	1,739 (60.4)	251 (8.7)		985 (34.2)	944 (32.8)	949 (33.0)		
Median (Min-Max)^2^	3.0 (0.0-8.0)	15.0 (9.0-26.0)	30.0 (27.0-47.0)		4.0 (0.0-9.0)	13.0 (10.0-16.0)	22.0 (17.0-47.0)		
No. of cases/person-years	54/5,783	122/10,183	27/1,347		62/6,367	64/5,778	77/5,168		
Age-adjusted model	1.00 (reference)	1.33 (0.97, 1.83)	2.15 (1.37, 3.39)	0.001	1.00 (reference)	1.18 (0.84, 1.67)	1.58 (1.13, 2.20)	0.007	1.12 (1.04, 1.21)
Multivariable model 1^3^	1.00 (reference)	1.33 (0.97, 1.83)	2.15 (1.37, 3.39)	0.001	1.00 (reference)	1.18 (0.83, 1.67)	1.58 (1.13, 2.19)	0.007	1.12 (1.04, 1.21)
Multivariable model 2^3^	1.00 (reference)	1.24 (0.90, 1.72)	2.23 (1.41, 3.52)	0.002	1.00 (reference)	1.13 (0.80, 1.60)	1.50 (1.07, 2.10)	0.018	1.13 (1.04, 1.22)
Female									
Baseline									
n (%)	781 (15.6)	3,324 (66.5)	897 (17.9)		1,609 (32.2)	1,678 (33.6)	1,715 (34.3)		
Median (Min-Max)^2^	5.0 (0.0-8.0)	17.0 (9.0-26.0)	31.0 (27.0-54.0)		9.0 (0.0-13.0)	17.0 (14.0-21.0)	27.0 (22.0-54.0)		
No. of cases/person-years	29/4,283	159/20,589	46/5,436		71/9,322	80/10,337	83/10,650		
Age-adjusted model	1.00 (reference)	1.18 (0.79, 1.74)	1.25 (0.79, 1.98)	0.363	1.00 (reference)	1.03 (0.75, 1.42)	1.02 (0.75, 1.40)	0.907	1.02 (0.95, 1.10)
Multivariable model 1^3^	1.00 (reference)	1.18 (0.79, 1.74)	1.24 (0.78, 1.97)	0.387	1.00 (reference)	1.03 (0.75, 1.42)	1.01 (0.74, 1.39)	0.951	1.02 (0.95, 1.10)
Multivariable model 2^3^	1.00 (reference)	1.30 (0.88, 1.93)	1.35 (0.85, 2.17)	0.236	1.00 (reference)	1.09 (0.79, 1.50)	1.07 (0.78, 1.48)	0.704	1.04 (0.97, 1.11)
Cumulative average									
n (%)	687 (13.7)	3,657 (73.1)	658 (13.2)		1,685 (33.7)	1,601 (32.0)	1,716 (34.3)		
Median (Min-Max)^2^	5.0 (0.0-8.0)	16.3 (8.3-26.7)	30.0 (27.0-53.0)		9.0 (0.0-13.0)	16.0 (13.3-19.7)	25.0 (20.0-53.0)		
No. of cases/person-years	25/3,688	175/23,105	34/3,516		69/9,920	70/10,447	95/9,941		
Age-adjusted model	1.00 (reference)	1.15 (0.76, 1.75)	1.43 (0.86, 2.39)	0.086	1.00 (reference)	0.98 (0.70, 1.36)	1.39 (1.02, 1.89)	0.028	1.08 (1.00, 1.17)
Multivariable model 1^3^	1.00 (reference)	1.15 (0.76, 1.75)	1.42 (0.85, 2.37)	0.168	1.00 (reference)	0.98 (0.70, 1.36)	1.38 (1.02, 1.89)	0.030	1.08 (1.00, 1.17)
Multivariable model 2^3^	1.00 (reference)	1.28 (0.84, 1.94)	1.61 (0.95, 2.74)	0.072	1.00 (reference)	1.01 (0.72, 1.41)	1.46 (1.07, 2.01)	0.013	1.10 (1.02, 1.19)
Recent									
n (%)	1,184 (23.7)	3,150 (63.0)	668 (13.4)		1,724 (34.5)	1,655 (33.1)	1,623 (32.5)		
Median (Min-Max)^2^	4.0 (0.0-8.0)	16.0 (9.0-26.0)	31.0 (27.0-53.0)		6.0 (0.0-11.0)	15.0 (12.0-19.0)	25.0 (20.0-53.0)		
No. of cases/person-years	47/7,767	151/18,951	36/3,590		68/11,170	76/10,050	90/9,088		
Age-adjusted model	1.00 (reference)	1.35 (0.98, 1.87)	1.66 (1.08, 2.55)	0.015	1.00 (reference)	1.27 (0.91, 1.75)	1.65 (1.20, 2.25)	0.002	1.09 (1.03, 1.17)
Multivariable model 1^3^	1.00 (reference)	1.35 (0.98, 1.88)	1.65 (1.08, 2.54)	0.016	1.00 (reference)	1.27 (0.91, 1.76)	1.64 (1.20, 2.25)	0.002	1.09 (1.03, 1.17)
Multivariable model 2^3^	1.00 (reference)	1.43 (1.03, 1.98)	1.72 (1.11, 2.66)	0.008	1.00 (reference)	1.33 (0.96, 1.84)	1.73 (1.26, 2.37)	0.001	1.10 (1.03, 1.18)

All values were adjusted for age (otherwise specified) and expressed as mean±standard error for continuous variables or percentages for categorical variables; Mean values with different superscripts (a, b, c) within a row are significantly different between groups, according to Tukey’s multiple comparison test in the general linear model. PWI-SF, Psychosocial Well-Being Index-Short Form; Min, minimum; Max, maximum; FBG, fasting blood glucose.

1 Linear trends were obtained by treating the median value of each group as a continuous variable.

2 PWI-SF score observed within each stress-level group or tertile.

3 Multivariable model 1: adjusted for age and education; Multivariable model 2: adjusted for age, education, regular exercise, smoking status (current/former/never for male, and yes/no for female), alcohol consumption, body mass index, and Diet Quality Index-International.

**Table 3.** Sensitivity analysis of the associations between PWI-SF and the risk of T2D^1^

Variables	Categories of PWI-SF scores			p-trend^2^	Tertiles of PWI-SF scores			p-trend^2^
	Healthy group (PWI-SF≤8.0)	Potential stress group (8.0<PWI-SF<27.0)	High-risk group (PWI-SF≥27.0)		T1	T2	T3	
Male								
Baseline								
After excluding patients with CVD/cancer during follow-up	1.00 (reference)	0.89 (0.61, 1.30)	0.94 (0.54, 1.64)	0.758	1.00 (reference)	1.11 (0.78, 1.59)	0.73 (0.49, 1.09)	0.098
After excluding incident cases within the first year	1.00 (reference)	0.95 (0.67, 1.37)	1.13 (0.68, 1.89)	0.705	1.00 (reference)	1.13 (0.81, 1.57)	0.74 (0.51, 1.07)	0.086
Among those who visited two or more times	1.00 (reference)	1.15 (0.68, 1.96)	1.19 (0.58, 2.48)	0.625	1.00 (reference)	1.25 (0.78, 2.00)	0.95 (0.58, 1.57)	0.737
Among those who were married	1.00 (reference)	0.93 (0.64, 1.34)	1.06 (0.61, 1.83)	0.932	1.00 (reference)	1.15 (0.82, 1.63)	0.79 (0.54, 1.16)	0.188
Additionally adjusting baseline FBG	1.00 (reference)	0.94 (0.67, 1.34)	1.26 (0.76, 2.09)	0.468	1.00 (reference)	1.16 (0.84, 1.59)	0.87 (0.61, 1.24)	0.396
Excluding prediabetes participants at baseline	1.00 (reference)	1.47 (0.62, 3.52)	2.34 (0.86, 6.32)	0.073	1.00 (reference)	1.00 (0.47, 2.16)	1.48 (0.72, 3.01)	0.230
Additionally adjusting cohort (fixed effect)	1.00 (reference)	0.97 (0.68, 1.38)	1.17 (0.71, 1.92)	0.617	1.00 (reference)	1.15 (0.83, 1.60)	0.78 (0.54, 1.12)	0.147
Multivariable model (-BMI)	1.00 (reference)	0.97 (0.68, 1.38)	1.10 (0.67, 1.81)	0.749	1.00 (reference)	1.15 (0.82, 1.60)	0.75 (0.52, 1.07)	0.079
T2D diagnosis based on medication treatment only	1.00 (reference)	0.81 (0.53, 1.23)	1.05 (0.58, 1.91)	0.987	1.00 (reference)	1.03 (0.68, 1.54)	0.74 (0.48, 1.16)	0.173
Cumulative average								
After excluding patients with CVD/cancer during follow-up	1.00 (reference)	0.86 (0.60, 1.24)	1.67 (0.93, 2.97)	0.287	1.00 (reference)	0.99 (0.68, 1.42)	1.06 (0.73, 1.54)	0.756
After excluding incident cases within the first year	1.00 (reference)	0.97 (0.68, 1.39)	2.00 (1.17, 3.40)	0.054	1.00 (reference)	0.99 (0.70, 1.41)	1.07 (0.75, 1.52)	0.698
Among those who visited two or more times	1.00 (reference)	1.22 (0.72, 2.07)	1.95 (0.86, 4.40)	0.141	1.00 (reference)	1.18 (0.73, 1.89)	1.11 (0.68, 1.84)	0.673
Among those who were married	1.00 (reference)	0.97 (0.67, 1.41)	2.14 (1.23, 3.72)	0.043	1.00 (reference)	1.08 (0.76, 1.54)	1.11 (0.77, 1.60)	0.587
Additionally adjusting baseline FBG	1.00 (reference)	1.07 (0.75, 1.51)	2.15 (1.27, 3.63)	0.017	1.00 (reference)	1.12 (0.81, 1.54)	1.25 (0.90, 1.75)	0.184
Excluding prediabetes participants at baseline	1.00 (reference)	1.47 (0.61, 3.51)	3.39 (1.17, 9.88)	0.019	1.00 (reference)	0.96 (0.44, 2.10)	1.77 (0.87, 3.57)	0.077
Additionally adjusting cohort (fixed effect)	1.00 (reference)	0.97 (0.68, 1.38)	2.08 (1.24, 3.50)	0.034	1.00 (reference)	1.02 (0.73, 1.44)	1.11 (0.78, 1.56)	0.564
Multivariable model (-BMI)	1.00 (reference)	0.93 (0.65, 1.31)	1.84 (1.09, 3.10)	0.085	1.00 (reference)	1.02 (0.72, 1.43)	1.05 (0.74, 1.48)	0.777
T2D diagnosis based on medication treatment only	1.00 (reference)	0.81 (0.53, 1.24)	1.94 (1.04, 3.62)	0.185	1.00 (reference)	0.84 (0.55, 1.27)	0.89 (0.58, 1.37)	0.596
Recent								
After excluding patients with CVD/cancer during follow-up	1.00 (reference)	1.17 (0.83, 1.65)	1.85 (1.09, 3.14)	0.045	1.00 (reference)	1.12 (0.77, 1.62)	1.36 (0.94, 1.96)	0.101
After excluding incident cases within the first year	1.00 (reference)	1.22 (0.88, 1.70)	2.15 (1.34, 3.43)	0.005	1.00 (reference)	1.12 (0.78, 1.59)	1.44 (1.02, 2.02)	0.039
Among those who visited two or more times	1.00 (reference)	1.08 (0.70, 1.68)	1.96 (1.02, 3.78)	0.124	1.00 (reference)	1.02 (0.63, 1.63)	1.21 (0.76, 1.93)	0.438
Among those who were married	1.00 (reference)	1.22 (0.80, 1.71)	2.16 (1.31, 3.55)	0.008	1.00 (reference)	1.15 (0.80, 1.65)	1.45 (1.02, 2.05)	0.041
Additionally adjusting baseline FBG	1.00 (reference)	1.25 (0.92, 1.70)	2.28 (1.45, 3.60)	0.001	1.00 (reference)	1.25 (0.89, 1.75)	1.57 (1.13, 2.18)	0.007
Excluding prediabetes participants at baseline	1.00 (reference)	1.50 (0.75, 2.97)	3.09 (1.30, 7.37)	0.015	1.00 (reference)	1.16 (0.54, 2.46)	2.02 (1.03, 3.95)	0.034
Additionally adjusting cohort (fixed effect)	1.00 (reference)	1.22 (0.89, 1.68)	2.20 (1.40, 3.45)	0.003	1.00 (reference)	1.12 (0.79, 1.59)	1.47 (1.06, 2.05)	0.023
Multivariable model (-BMI)	1.00 (reference)	1.27 (0.92, 1.76)	2.03 (1.28, 3.22)	0.005	1.00 (reference)	1.14 (0.80, 1.62)	1.50 (1.07, 2.09)	0.019
T2D diagnosis based on medication treatment only	1.00 (reference)	0.98 (0.66, 1.45)	2.14 (1.25, 3.67)	0.053	1.00 (reference)	0.85 (0.55, 1.33)	1.30 (0.86, 1.95)	0.234
Female								
Baseline								
After excluding patients with CVD/cancer during follow-up	1.00 (reference)	1.37 (0.90, 2.10)	1.35 (0.81, 2.25)	0.310	1.00 (reference)	1.13 (0.80, 1.59)	1.08 (0.76, 1.53)	0.713
After excluding incident cases within the first year	1.00 (reference)	1.25 (0.84, 1.86)	1.28 (0.80, 2.07)	0.344	1.00 (reference)	1.09 (0.79, 1.51)	1.09 (0.78, 1.51)	0.637
Among those who visited two or more times	1.00 (reference)	1.75 (0.97, 3.17)	2.22 (1.14, 4.33)	0.016	1.00 (reference)	1.35 (0.86, 2.10)	1.49 (0.96, 2.32)	0.083
Among those who were married	1.00 (reference)	1.25 (0.80, 1.94)	1.45 (0.86, 2.45)	0.158	1.00 (reference)	1.10 (0.77, 1.57)	1.13 (0.79, 1.62)	0.509
Additionally adjusting baseline FBG	1.00 (reference)	1.44 (0.98, 2.12)	1.61 (1.01, 2.57)	0.048	1.00 (reference)	1.17 (0.86, 1.61)	1.23 (0.90, 1.70)	0.206
Excluding prediabetes participants at baseline	1.00 (reference)	1.87 (0.85, 4.10)	1.58 (0.62, 4.00)	0.477	1.00 (reference)	1.42 (0.79, 2.56)	1.62 (0.89, 2.96)	0.119
Additionally adjusting cohort (fixed effect)	1.00 (reference)	1.31 (0.88, 1.94)	1.38 (0.86, 2.21)	0.203	1.00 (reference)	1.10 (0.80, 1.51)	1.09 (0.79, 1.50)	0.637
Multivariable model (-BMI)	1.00 (reference)	1.17 (0.79, 1.74)	1.24 (0.78, 1.99)	0.387	1.00 (reference)	1.03 (0.75, 1.42)	1.02 (0.73, 1.41)	0.939
T2D diagnosis based on medication treatment only	1.00 (reference)	1.55 (0.92, 2.61)	2.06 (1.15, 3.69)	0.011	1.00 (reference)	1.44 (0.96, 2.17)	1.46 (0.97, 2.18)	0.083
Cumulative average								
After excluding patients with CVD/cancer during follow-up	1.00 (reference)	1.37 (0.86, 2.18)	1.62 (0.91, 2.89)	0.101	1.00 (reference)	1.03 (0.72, 1.47)	1.46 (1.04, 2.05)	0.022
After excluding incident cases within the first year	1.00 (reference)	1.24 (0.81, 1.89)	1.50 (0.88, 2.57)	0.136	1.00 (reference)	1.01 (0.72, 1.42)	1.50 (1.09, 2.07)	0.010
Among those who visited two or more times	1.00 (reference)	2.32 (1.14, 4.73)	3.04 (1.33, 6.95)	0.005	1.00 (reference)	1.42 (0.90, 2.25)	2.05 (1.31, 3.20)	0.001
Among those who were married	1.00 (reference)	1.20 (0.75, 1.93)	1.82 (1.02, 3.23)	0.034	1.00 (reference)	1.04 (0.72, 1.51)	1.55 (1.08, 2.21)	0.012
Additionally adjusting baseline FBG	1.00 (reference)	1.30 (0.87, 1.96)	1.88 (1.12, 3.13)	0.015	1.00 (reference)	1.14 (0.82, 1.59)	1.58 (1.15, 2.15)	0.003
Excluding prediabetes participants at baseline	1.00 (reference)	1.49 (0.66, 3.36)	2.06 (0.79, 5.37)	0.120	1.00 (reference)	1.35 (0.73, 2.50)	2.06 (1.13, 3.75)	0.012
Additionally adjusting cohort (fixed effect)	1.00 (reference)	1.29 (0.84, 1.96)	1.65 (0.98, 2.79)	0.057	1.00 (reference)	1.01 (0.73, 1.41)	1.48 (1.08, 2.02)	0.011
Multivariable model (-BMI)	1.00 (reference)	1.15 (0.76, 1.76)	1.44 (0.85, 2.43)	0.167	1.00 (reference)	0.98 (0.70, 1.37)	1.39 (1.01, 1.92)	0.031
T2D diagnosis based on medication treatment only	1.00 (reference)	1.68 (0.95, 2.96)	2.21 (1.13, 4.29)	0.016	1.00 (reference)	1.30 (0.86, 1.95)	1.76 (1.18, 2.62)	0.004
Recent								
After excluding patients with CVD/cancer during follow-up	1.00 (reference)	1.46 (1.02, -2.08)	1.70 (1.06, 2.71)	0.015	1.00 (reference)	1.32 (0.93, 1.87)	1.72 (1.23, 2.41)	0.001
After excluding incident cases within the first year	1.00 (reference)	1.38 (0.99, -1.91)	1.60 (1.02, 2.49)	0.024	1.00 (reference)	1.23 (0.88, 1.71)	1.68 (1.23, 2.31)	0.001
Among those who visited two or more times	1.00 (reference)	1.29 (0.85, -1.95)	1.79 (1.03, 3.11)	0.039	1.00 (reference)	1.38 (0.90, 2.13)	1.91 (1.27, 2.89)	0.002
Among those who were married	1.00 (reference)	1.42 (0.98, -2.07)	2.11 (1.32, 3.37)	0.002	1.00 (reference)	1.41 (0.98, 2.03)	1.79 (1.25, 2.55)	0.001
Additionally adjusting baseline FBG	1.00 (reference)	1.45 (1.05, -1.99)	1.62 (1.05, 2.51)	0.017	1.00 (reference)	1.34 (0.97, 1.86)	1.76 (1.29, 2.39)	<0.001
Excluding prediabetes participants at baseline	1.00 (reference)	1.57 (0.87, -2.82)	1.75 (0.82, 3.74)	0.104	1.00 (reference)	1.56 (0.87, 2.80)	2.11 (1.21, 3.68)	0.007
Additionally adjusting cohort (fixed effect)	1.00 (reference)	1.42 (1.03, -1.97)	1.73 (1.13, 2.67)	0.007	1.00 (reference)	1.33 (0.96, 1.84)	1.73 (1.27, 2.37)	<0.001
Multivariable model (-BMI)	1.00 (reference)	1.35 (0.97, -1.87)	1.67 (1.08, 2.58)	0.016	1.00 (reference)	1.27 (0.92, 1.76)	1.65 (1.20, 2.26)	0.002
T2D diagnosis based on medication treatment only	1.00 (reference)	1.57 (1.04, -2.38)	2.08 (1.25, 3.49)	0.003	1.00 (reference)	1.38 (0.92, 2.08)	1.95 (1.33, 2.85)	<0.001

PWI-SF, Psychosocial Well-Being Index-Short Form; T2D, type 2 diabetes; CVD, cardiovascular disease; FBG, fasting blood glucose; BMI, body mass index.

1 Multivariable model: adjusted for age, education level, regular exercise, smoking status (current/former/never for male, and yes/no for female), alcohol consumption, BMI, and Diet Quality Index – International.

2 p-values for linear trends were obtained by treating the median value of each group as a continuous variable.

## Supplementary materials

Supplementary Material 1. Flowchart of study in KoGES_CAVAS cohort

Supplementary Material 2. Evidence-based directed acyclic graph (DAG) for prospective associations between psychosocial stress and type 2 diabetes development https://dagitty.net/mWXJJFWxx.

Supplementary Material 3. STROBE Statement for cohort studies

Supplementary Material 4. Baseline characteristics of the study participants

Supplementary Material 5. Age and sex adjusted baseline characteristics of the study participants (included vs. excluded)

Supplementary Material 6. Kaplan-Meier probability curve for incident type 2 diabetes according to psychosocial stress level in men and women.

Supplementary Material 7. Age-adjusted characteristics of study participants according to psychosocial stress levels measured using the Psychosocial Well-being Index-Short Form (PWI-SF) for men and women

Supplementary Material 8. Multivariate-adjusted incidence rate ratio of type 2 diabetes risk according to psychosocial stress level (PWI-SF score) and covariates in men.

Supplementary Material 9. Stratified analyses of the association between Psychosocial Well-being Index-short Form (PWI-SF) scores and type 2 diabetes risk in women

Supplementary Material 10.

Supplementary Material 11. Incidence rate ratios of type 2 diabetes by psychosocial stress levels (baseline, cumulative average, and recent PWI-SF scores) from multiple imputation analysis

Supplementary Material 12. Methods for multiple imputation
