# Supplementary material for: Prospective associations between psychosocial stress and the risk of type 2 diabetes in middle-aged adults: findings from the KoGES_CAVAS
Source: Epidemiol Health. 2025 Oct 31;47:e2025061. doi: 10.4178/epih.e2025061 (PMC12885608; doi:10.4178/epih.e2025061)
Supplement: Supplementary Material 1. — Flowchart of study in KoGES_CAVAS cohort [file epih-47-e2025061-Supplementary-1.docx]

n=15,701

n=10,604

n=9,514

**n=7,880**

**men 2,878 (36.5%); women 5,002 (63.5%)**

Excluded participants aged ≥ 65-year-old
(n=5,097, 32.5%)

Excluded participants with T2D (n=1,042, 9.83%) or

missing information on baseline T2D status (n=48, 0.45%)

Excluded participants with missing baseline PWI-SF (n=1,288, 13.5%)

**KoGES_CAVAS n=19,546**

Excluded Kangwha cohort participants
due to no PWI-SF assessment
 (n = 3,845, 19.7%)

n=8,226

Excluded participants with missing data for key covariates
(education level, regular exercise, smoking status, physical activity, drinking status, BMI) (n=346, 4.2%)

**Supplementary Material. 1.** Flowchart of study in KoGES_CAVAS cohort

The percentage for each exclusion criteria was calculated from the number of participants remaining after the preceding step.
