# Supplementary material for: Prospective associations between psychosocial stress and the risk of type 2 diabetes in middle-aged adults: findings from the KoGES_CAVAS
Source: Epidemiol Health. 2025 Oct 31;47:e2025061. doi: 10.4178/epih.e2025061 (PMC12885608; doi:10.4178/epih.e2025061)
Supplement: Supplementary Material 2. — Evidence-based directed acyclic graph (DAG) for prospective associations between psychosocial stress and type 2 diabetes development https://dagitty.net/mWXJJFWxx. [file epih-47-e2025061-Supplementary-2.docx]

**
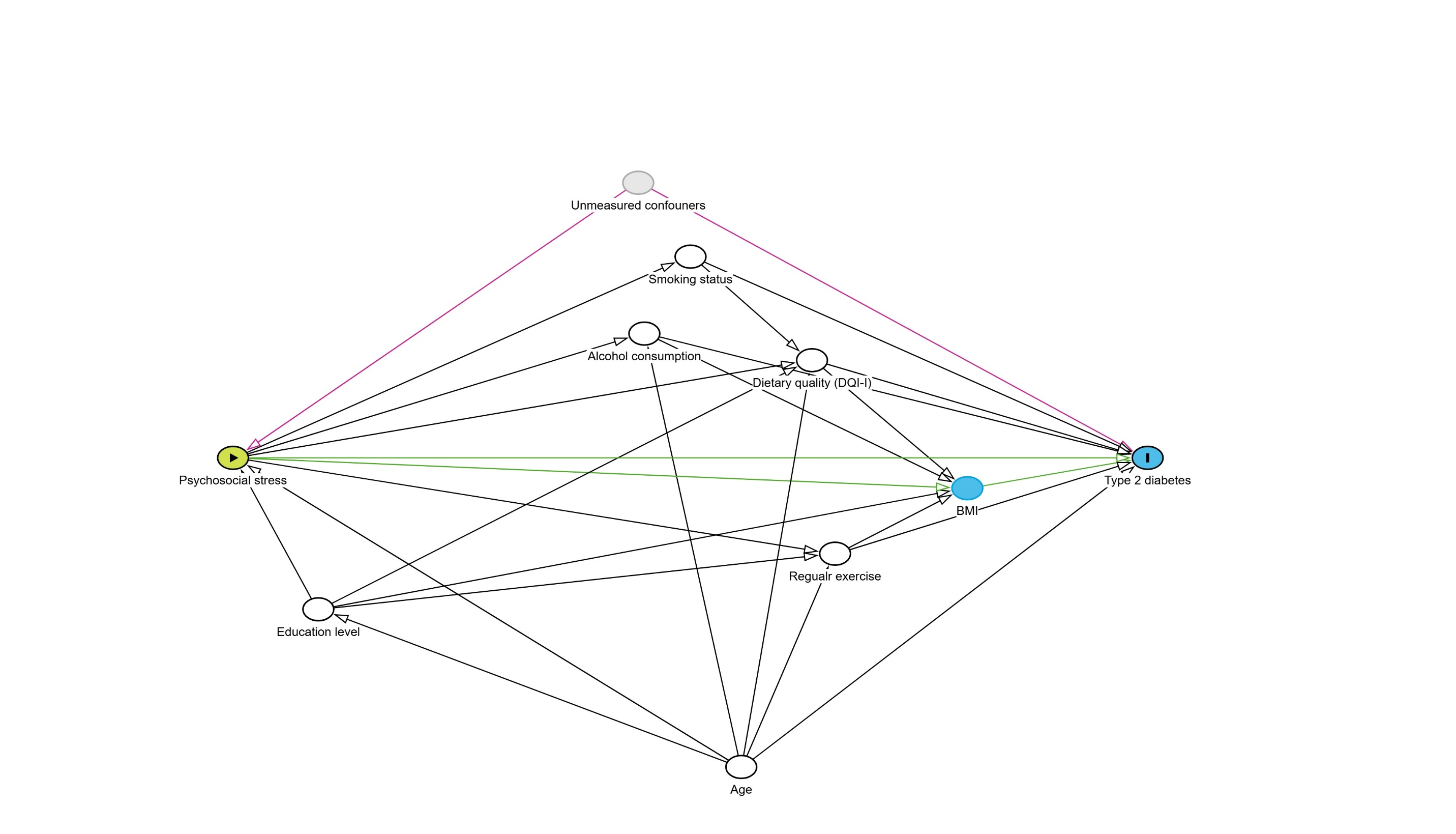
**

**Supplementary Material. 2.** Evidence-based directed acyclic graph (DAG) for prospective associations between psychosocial stress and type 2 diabetes development <https://dagitty.net/mWXJJFWxx>.
