# Supplementary material for: Prospective associations between psychosocial stress and the risk of type 2 diabetes in middle-aged adults: findings from the KoGES_CAVAS
Source: Epidemiol Health. 2025 Oct 31;47:e2025061. doi: 10.4178/epih.e2025061 (PMC12885608; doi:10.4178/epih.e2025061)
Supplement: Supplementary Material 4. — Baseline characteristics of the study participants [file epih-47-e2025061-Supplementary-4.docx]

**Supplementary Material 4.** Baseline characteristics of the study participants

| **Characteristics** | **Total** | **Men** | **Women** | ***p*-value^1^** |
| --- | --- | --- | --- | --- |
| n (%) | 7,880 (100.0) | 2,878 (36.5) | 5,002 (63.5) |  |
| Age, years | 53.1 ± 6.7 | 53.8 ± 6.7 | 52.7 ± 6.7 | <0.0001 |
| Higher education, n^2^ (%) | 2,959 (37.6) | 1,336 (46.4) | 1,623 (32.5) | <0.0001 |
| Regular exercise, n^3^ (%) | 1,873 (23.8) | 602 (20.9) | 1,271 (25.4) | <0.0001 |
| Smoking status, n (%) |  |  |  |  |
| Never smoker | 5,707 (72.4) | 825 (28.7) | 4,882 (97.6) | <.0001 |
| Past smoker | 965 (12.3) | 937 (32.6) | 28 (0.56) |  |
| Current smoker | 1,208 (15.3) | 1,116 (38.8) | 92 (1.84) |  |
| Current drinker, n (%) | 3,597 (45.7) | 2,041 (70.9) | 1,556 (31.1) | <0.0001 |
| Alcohol consumption, g/day | 10.3 ± 28.1 | 24.3 ± 40.8 | 2.2 ±10.2 | <0.0001 |
| Body mass index, kg/m^2^ | 24.5 ± 3.1 | 24.5 ± 2.9 | 24.6 ± 3.2 | 0.4900 |
| Waist circumference, cm | 83.4 ± 8.7 | 86.3 ± 7.9 | 81.7 ± 8.7 | <0.0001 |
| Fasting blood glucose, mg/dL | 93.6 ± 9.8 | 96.2 ± 10.3 | 92.1 ± 9.2 | <0.0001 |
| Menopausal status, % | - | - | 3,232 (65.2) | - |
| Psychosocial Well-being Index Short Form (PWI-SF) score | | | |  |
| Baseline score | 17.2 ± 9.1 | 15.7 ± 8.6 | 18.1 ± 9.3 | <0.0001 |
| Cumulative average score | 16.2 ± 8.1 | 14.6 ± 7.7 | 17.1 ± 8.3 | <0.0001 |
| Recent score (most recent score before the diagnosis) | 14.9 ± 9.3 | 13.4 ± 8.8 | 15.7 ± 9.5 | <0.0001 |

Values are expressed as mean±SD for continuous variables and numbers and percentages for categorical variables.

^1^ *p*-values were obtained using independent t-tests between men and women.

^2^ Higher education level (≥12 years of education).

^3^ Regular exercise (≥3 times/week and ≥30 min/session).
