# Supplementary material for: Prospective associations between psychosocial stress and the risk of type 2 diabetes in middle-aged adults: findings from the KoGES_CAVAS
Source: Epidemiol Health. 2025 Oct 31;47:e2025061. doi: 10.4178/epih.e2025061 (PMC12885608; doi:10.4178/epih.e2025061)
Supplement: Supplementary Material 5. — Age and sex adjusted baseline characteristics of the study participants (included vs. excluded) [file epih-47-e2025061-Supplementary-5.docx]

**Supplementary Material 5.** Age and sex adjusted baseline characteristics of the study participants (included vs. excluded)^1^

| **Characteristic** | **Included Particpants** | **Excluded Particpants** | **p-value^2^** |
| --- | --- | --- | --- |
| n (%) | 7,880 (82.4) | 1,682 (17.6) |  |
| Men, n (%) | 36.7^b^ | 29.6^a^ | <.0001 |
| Age, y | 53.1±0.1 | 55.4±0.2 | <.0001 |
| Higher education, n^3^ (%) | 36.4^b^ | 24.1^a^ | <.0001 |
| Regular exercise, n^4^ (%) | 23.8^b^ | 17.8^a^ | <.0001 |
| Smoking statuis, n (%) | 14.8^a^ | 17.9^b^ | 0.0003 |
| Current drinker, n (%) | 44.9^a^ | 47.7^b^ | 0.0262 |
| Alcohol consumption, g/day | 9.9±0.3 | 10.7±0.7 | 0.0665 |
| Body Mass Index (BMI), kg/m2 | 24.6±0.0 | 24.7±0.1 | 0.0744 |
| Waist Circumference (WC), cm | 83.4±0.1 | 83.7±0.2 | 0.2452 |
| Fasting Blood Glucose, mg/dL | 93.6±0.1 | 93.8±0.2 | 0.4460 |

^1^ All values were adjusted for age and sex and expressed as mean ± SE for continuous variables or percentages for categorical variables. Mean values with different Superscripts (a, b, c) within a row were significantly different between groups by Tukey's multiple comparison test.
^2^ P values for differences were determined by the General Linear Model (Tukey's multiple comparison).
^3^ Higher education level (≥ 12 years of education).
^4^ Regular exercise (≥ 3 times/week and ≥ 30 min/session)
