# Supplementary material for: Prospective associations between psychosocial stress and the risk of type 2 diabetes in middle-aged adults: findings from the KoGES_CAVAS
Source: Epidemiol Health. 2025 Oct 31;47:e2025061. doi: 10.4178/epih.e2025061 (PMC12885608; doi:10.4178/epih.e2025061)
Supplement: Supplementary Material 6. — Kaplan-Meier probability curve for incident type 2 diabetes according to psychosocial stress level in men and women. [file epih-47-e2025061-Supplementary-6.docx]

**Supplementary Material 6.** Kaplan-Meier probability curve for incident type 2 diabetes according to psychosocial stress level in men and women.


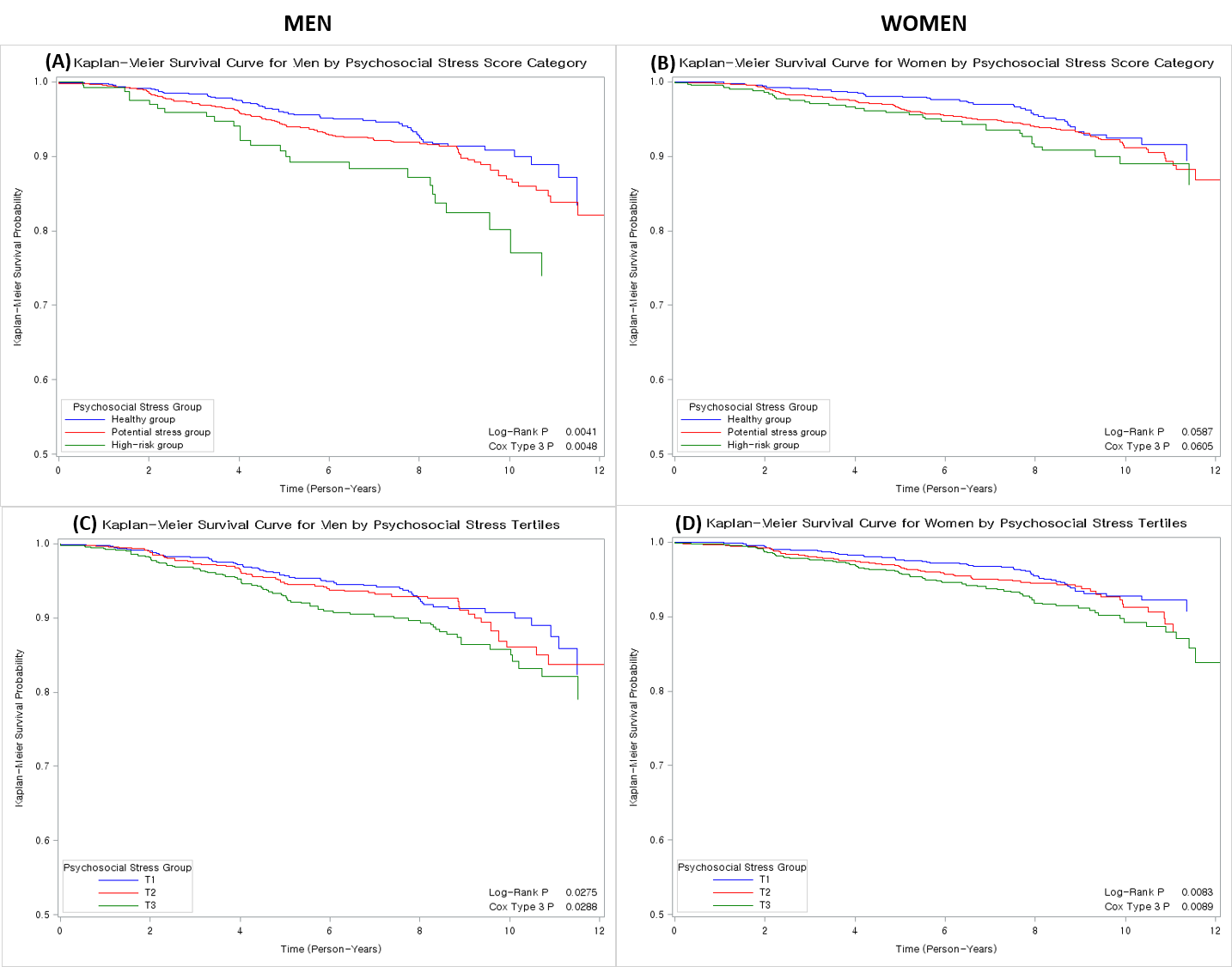


Kaplan-Meier curves illustrate the probability of remaining the non-case of type 2 diabetes over the follow-up period. The analyses are presented for men, stratified by (A) psychosocial stress score category and (B) stress score tertiles, and for women, stratified by (C) psychosocial stress score category and (D) stress score tertiles. P-values were derived from the log-rank test comparing the survival distributions among the groups.
