# Supplementary material for: Prospective associations between psychosocial stress and the risk of type 2 diabetes in middle-aged adults: findings from the KoGES_CAVAS
Source: Epidemiol Health. 2025 Oct 31;47:e2025061. doi: 10.4178/epih.e2025061 (PMC12885608; doi:10.4178/epih.e2025061)
Supplement: Supplementary Material 7. — Age-adjusted characteristics of study participants according to psychosocial stress levels measured using the Psychosocial Well-being Index-Short Form (PWI-SF) for men and women [file epih-47-e2025061-Supplementary-7.docx]

**Supplementary Material 7.** Age-adjusted characteristics of study participants according to psychosocial stress levels measured using the Psychosocial Well-being Index-Short Form (PWI-SF) for men and women

|  | **Categories of PWI-SF scores** | | | | | | ***p-*trend^1^** | | **Tertiles of PWI-SF scores** | | | | | | ***p-*trend^1^** | |
| --- | --- | --- | --- | --- | --- | --- | --- | --- | --- | --- | --- | --- | --- | --- | --- | --- |
|  | **Healthy group** | | **Potential stress group** | | | **Stress group** |  |  | **T1** | | **T2** | | **T3** | |  |  |
|  | **(PWI-SF≤8.0)** | | **(8.0<PWI-SF<27.0)** | | | **(PWI-SF≥27.0)** |  |  |  |  |  |  |  |  |  |  |
| **MEN** |  | |  | | |  |  | |  | |  | |  | |  | |
| **CUMULATIVE AVERAGE** | | | |  | |  |  | |  | |  | |  | |  | |
| n (%) | 593 (20.6) | | 2,073 (72.0) | | | 212 (7.4) |  | | 995 (34.6) | | 937 (32.6) | | 946 (32.9) | |  | |
| Median (min-max)^2^ | 5.0 (0.0‒8.0) | | 15.0 (8.3‒26.7) | | | 30.0 (27.0‒47.0) |  | | 7.3 (0.0‒11.0) | | 14.0 (11.3‒17.0) | | 22.0 (17.3‒47.0) | |  | |
| Age, years | 55.1±0.3b | | 53.4±0.1a | | | 53.8±0.5a | 0.0002 | | 54.6±0.2^a^ | | 53.2±0.2^b^ | | 53.5±0.2^b^ | | 0.0003 | |
| Higher education, %^3^ | 45.1^b^ | | 48.0^b^ | | | 34.9^a^ | 0.0616 | | 47.2^a^ | | 51.7^a^ | | 40.4^b^ | | 0.0009 | |
| Regular exercise, %^4^ | 25.9^c^ | | 20.8^b^ | | | 8.5^a^ | <0.0001 | | 25.2^a^ | | 22.8^a^ | | 14.6^b^ | | <0.0001 | |
| Current smoker, % | 31.9^a^ | | 39.5^b^ | | | 50.9^c^ | <0.0001 | | 32.6^b^ | | 39.9^a^ | | 44.2^a^ | | <0.0001 | |
| Current drinker, % | 68.4 | | 72.1 | | | 66.5 | 0.9491 | | 69.0 | | 71.1 | | 72.8 | | 0.0680 | |
| Alcohol consumption, g/day | 23.1±1.7 | | 24.3±0.9 | | | 27.4±2.8 | 0.2043 | | 23.7±1.3 | | 22.5±1.3 | | 26.7±1.3 | | 0.0908 | |
| Body mass index, kg/m^2^ | 24.8±0.1^b^ | | 24.5±0.1^b^ | | | 24.0±0.2^a^ | 0.0003 | | 24.7±0.1^a^ | | 24.6±0.1^a^ | | 24.2±0.1^b^ | | <0.0001 | |
| Waist circumference, cm | 86.8±0.3^b^ | | 86.2±0.2^ab^ | | | 85.2±0.5^a^ | 0.0101 | | 86.9±0.3^a^ | | 86.4±0.3^a^ | | 85.6±0.3^b^ | | 0.0002 | |
| Fasting blood glucose, mg/dL | 96.7±0.4 | | 96.1±0.2 | | | 95.3±0.7 | 0.0665 | | 96.3±0.3 | | 96.3±0.3 | | 95.8±0.3 | | 0.3267 | |
|  |  | |  | | |  |  | |  | |  | |  | |  | |
| **RECENT** |  | |  | | |  |  | |  | |  | |  | |  | |
| n (%) | 888 (30.9) | | 1,739 (60.4) | | | 251 (8.7) |  | | 985 (34.2) | | 944 (32.8) | | 949 (33.0) | |  | |
| Median (min-max)^2^ | 3.0 (0.0‒8.0) | | 15.0 (9.0‒26.0) | | | 30.0 (27.0‒47.0) |  | | 4.0 (0.0‒9.0)` | | 13.0 (10.0‒16.0) | | 22.0 (17.0‒47.0) | |  | |
| Age, years | 54.8±0.2^a^ | | 53.2±0.2^b^ | | | 54.3±0.4^a^ | 0.0018 | | 54.7±0.2^a^ | | 53.2±0.2^b^ | | 53.4±0.2^b^ | | <0.0001 | |
| Higher Education, %^3^ | 41.4^b^ | | 50.4^a^ | | | 37.0^b^ | 0.3652 | | 42.9^b^ | | 53.1^a^ | | 43.4^b^ | | 0.8151 | |
| Regular exercise, %^4^ | 24.3^a^ | | 20.5^a^ | | | 11.8^b^ | <0.0001 | | 24.7^a^ | | 20.9^ab^ | | 17.0^b^ | | <0.0001 | |
| Current smoker, % | 31.7^c^ | | 40.9^b^ | | | 48.6^a^ | <0.0001 | | 32.3^c^ | | 39.2^b^ | | 45.1^a^ | | <0.0001 | |
| Current drinker, % | 69.2 | | 71.8 | | | 70.4 | 0.3922 | | 69.7 | | 70.1 | | 73.0 | | 0.1073 | |
| Alcohol consumption, g/day | 23.0±1.4 | | 24.5±1.0 | | | 27.3±2.6 | 0.1335 | | 23.0±1.3 | | 22.9±1.3 | | 27.0±1.3 | | 0.0347 | |
| Body mass index, kg/m^2^ | 24.6±0.1^a^ | | 24.5±0.1^a^ | | | 24.0±0.2^b^ | 0.0123 | | 24.6±0.1^a^ | | 24.6±0.1^ab^ | | 24.3±0.1^b^ | | 0.0139 | |
| Waist circumference, cm | 86.5±0.3 | | 86.3±0.2 | | | 85.2±0.5 | 0.0359 | | 86.6±0.3 | | 86.4±0.3 | | 85.9±0.3 | | 0.0473 | |
| Fasting blood glucose, mg/dL | 96.1±0.3 | | 96.3±0.2 | | | 95.4±0.6 | 0.5374 | | 96.3±0.3 | | 95.8±0.3 | | 96.4±0.3 | | 0.9017 | |
|  |  | |  | | |  |  | |  | |  | |  | |  | |
| **WOMEN** | |  | | |  |  | |  | |  | |  | |  | |  |
| **CUMULATIVE AVERAGE** | | | | |  |  | |  | |  | |  | |  | |  |
| n (%) | | 687 (13.7) | | | 3,657 (73.1) | 658 (13.2) | |  | | 1,685 (33.7) | | 1,601 (32.0) | | 1,716 (34.3) | |  |
| Median (min-max)^2^ | | 5.0 (0.0‒8.0) | | | 16.3 (8.3‒26.7) | 30.0 (27.0‒53.0) | |  | | 9.0 (0.0‒13.0) | | 16.0 (13.3‒19.7) | | 25.0 (20.0‒53.0) | |  |
| Age, years | | 53.5±0.3^b^ | | | 52.4±0.1^a^ | 53.2±0.3^b^ | | 0.7252 | | 53.0±0.2^a^ | | 52.4±0.2^b^ | | 52.6±0.2^ab^ | | 0.1965 |
| Higher Education, %^3^ | | 37.9^c^ | | | 33.5^b^ | 20.6^a^ | | <0.0001 | | 37.0^a^ | | 35.4^a^ | | 25.2^b^ | | <0.0001 |
| Regular exercise, %^4^ | | 37.6^c^ | | | 25.1^b^ | 14.5^a^ | | <0.0001 | | 33.9^a^ | | 24.5^b^ | | 17.9^c^ | | <0.0001 |
| Current smoker, % | | 1.0^a^ | | | 1.7^a^ | 3.5^b^ | | 0.0005 | | 1.42 | | 1.75 | | 2.33 | | 0.0465 |
| Current drinker, % | | 32.4 | | | 30.6 | 32.6 | | 0.8438 | | 30.1 | | 31.8 | | 31.4 | | 0.4387 |
| Alcohol consumption, g/day | | 1.7±0.4 | | | 2.3±0.2 | 2.5±0.4 | | 0.1479 | | 2.1±0.2 | | 2.3±0.3 | | 2.3±0.2 | | 0.6912 |
| Body mass index, kg/m^2^ | | 24.8±0.1^b^ | | | 24.5±0.1^a^ | 24.6±0.1^ab^ | | 0.4051 | | 24.6±0.1 | | 24.5±0.1 | | 24.6±0.1 | | 0.4981 |
| Waist circumference, cm | | 82.5±0.3^b^ | | | 81.5±0.1^a^ | 82.2±0.3^ab^ | | 0.6963 | | 82.1±0.2 | | 81.4±0.2 | | 81.7±0.2 | | 0.2204 |
| Fasting blood glucose, mg/dL | | 92.8±0.3^b^ | | | 92.0±0.2^ab^ | 91.6±0.4^a^ | | 0.0180 | | 92.5±0.2 | | 91.7±0.2 | | 92.0±0.2 | | 0.1964 |
| Menopausal status, % | | 63.3^a^ | | | 65.1^ab^ | 68.2^b^ | | 0.0072 | | 63.5b | | 65.9ab | | 66.3a | | 0.0207 |
|  | |  | | |  |  | |  | |  | |  | |  | |  |
| **RECENT** | |  | | |  |  | |  | |  | |  | |  | |  |
| n (%) | | 1,184 (23.7) | | | 3,150 (63.0) | 668 (13.4) | |  | | 1,724 (34.5) | | 1,655 (33.1) | | 1,623 (32.5) | |  |
| Median (min-max)^2^ | | 4.0 (0.0‒8.0) | | | 16.0 (9.0 - 26.0) | 31.0 (27.0‒53.0) | |  | | 6.0 (0.0‒11.0) | | 15.0 (12.0‒19.0) | | 25.0 (20.0‒53.0) | |  |
| Age, years | | 53.3±0.2^a^ | | | 52.3±0.1^b^ | 53.1±0.3^a^ | | 0.1354 | | 53.1±0.2^a^ | | 52.3±0.2^b^ | | 52.6±0.2^b^ | | 0.0170 |
| Higher education, %^3^ | | 31.2^b^ | | | 35.0^a^ | 22.6^c^ | | 0.0009 | | 32.6^b^ | | 37.1^a^ | | 27.5^c^ | | 0.0006 |
| Regular exercise, %^4^ | | 31.5^a^ | | | 25.2^b^ | 15.8^c^ | | <.0001 | | 31.6^a^ | | 25.0^b^ | | 19.3^c^ | | <0.0001 |
| Current smoker, % | | 0.84^b^ | | | 2.00^a^ | 2.84^a^ | | 0.0014 | | 1.21^b^ | | 1.88^ab^ | | 2.47^a^ | | 0.0071 |
| Current drinker, % | | 31.3 | | | 30.9 | 31.8 | | 0.8637 | | 29.8 | | 32.0 | | 31.5 | | 0.2962 |
| Alcohol consumption, g/day | | 2.0±0.3 | | | 2.3±0.2 | 2.6±0.4 | | 0.2183 | | 2.2±0.2 | | 2.4±0.3 | | 2.1±0.3 | | 0.9395 |
| Body mass index, kg/m^2^ | | 24.7±0.1^a^ | | | 24.5±0.1^b^ | 24.7±0.1^ab^ | | 0.6452 | | 24.7±0.1 | | 24.5±0.1 | | 24.5±0.1 | | 0.1461 |
| Waist circumference (WC), cm | | 82.1±0.2^ab^ | | | 81.5±0.2^b^ | 82.3±0.3^a^ | | 0.8248 | | 82.0±0.2 | | 81.5±0.2 | | 81.7±0.2 | | 0.3220 |
| Fasting blood glucose, mg/dL | | 92.2±0.3 | | | 92.0±0.2 | 92.1±0.4 | | 0.7341 | | 92.1±0.2 | | 92.1±0.2 | | 92.1±0.2 | | 0.9861 |
| Menopausal status, % | | 64.0 | | | 65.4 | 66.6 | | 0.1119 | | 64.4 | | 64.8 | | 66.5 | | 0.0672 |

All values were adjusted for age (otherwise specified) and expressed as mean ± SE for continuous variables or percentages for categorical variables. Mean values with different superscripts (a, b, c) within a row are significantly different between groups, according to Tukey's multiple comparison test in the General Linear Model.

^1^ *p*-values for linear trends were obtained by treating the median value of each group as a continuous variable.

^2^ ‘Median (min-max)’ indicates the median and range (minimum-maximum) of the PWI-SF score observed within each stress-level group or tertile.

^3^ Higher education level (≥ 12 years of education).

^4^ Regular exercise (≥ 3 times/week and ≥ 30 min/session)
