# Supplementary material for: Prospective associations between psychosocial stress and the risk of type 2 diabetes in middle-aged adults: findings from the KoGES_CAVAS
Source: Epidemiol Health. 2025 Oct 31;47:e2025061. doi: 10.4178/epih.e2025061 (PMC12885608; doi:10.4178/epih.e2025061)
Supplement: Supplementary Material 8. — Multivariate-adjusted incidence rate ratio of type 2 diabetes risk according to psychosocial stress level (PWI-SF score) and covariates in men. [file epih-47-e2025061-Supplementary-8.docx]

**Supplementary Material 8.** Multivariate-adjusted incidence rate ratio of type 2 diabetes risk according to psychosocial stress level (PWI-SF score) and covariates in men.

|  | **Categories of PWI-SF scores** | | | ***p*-trend^1^** | ***p*-int^2^** | **Tertiles of PWI-SF scores** | | | ***p*-trend^1^** | ***p*-int^2^** |
| --- | --- | --- | --- | --- | --- | --- | --- | --- | --- | --- |
|  | **Healthy group** | **Potential stress group** | **High-risk group** |  |  | **T1** | **T2** | **T3** |  |  |
| **BASELINE** |  |  |  |  |  |  |  |  |  |  |
| **Level of Education** |  |  |  |  |  |  |  |  |  |  |
| <12 years | 1.00 | 0.83 (0.53–1.29) | 0.99 (0.54–1.82) | 0.8913 | 0.4097 | 1.00 | 1.26 (0.82–1.94) | 0.67 (0.42–1.07) | 0.0542 | 0.2699 |
| ≥12 years | 1.00 | 1.20 (0.65–2.19) | 1.45 (0.60–3.52) | 0.4047 |  | 1.00 | 1.03 (0.62–1.71) | 0.98 (0.55–1.74) | 0.9378 |  |
| **Regular Exercise** |  |  |  |  |  |  |  |  |  |  |
| No | 1.00 | 0.90 (0.60–1.33) | 1.00 (0.58–1.72) | 0.9665 | 0.4920 | 1.00 | 1.10 (0.76–1.58) | 0.71 (0.48–1.05) | 0.0582 | 0.3265 |
| Yes | 1.00 | 1.10 (0.48–2.49) | 2.25 (0.64–7.92) | 0.3591 |  | 1.00 | 1.39 (0.64–3.04) | 1.35 (0.58–3.15) | 0.4276 |  |
| **Current Smoker** |  |  |  |  |  |  |  |  |  |  |
| No | 1.00 | 0.89 (0.57–1.39) | 1.33 (0.69–2.55) | 0.6245 | 0.9786 | 1.00 | 1.05 (0.68–1.62) | 0.92 (0.57–1.46) | 0.7139 | 0.3765 |
| Yes | 1.00 | 1.05 (0.57–1.92) | 0.99 (0.45–2.17) | 0.9659 |  | 1.00 | 1.31 (0.78–2.19) | 0.64 (0.37–1.13) | 0.0563 |  |
| **Current Drinker** |  |  |  |  |  |  |  |  |  |  |
| No | 1.00 | 0.65 (0.37–1.15) | 1.00 (0.47–2.12) | 0.7891 | 0.4274 | 1.00 | 0.71 (0.38–1.34) | 0.75 (0.42–1.34) | 0.3568 | 0.7701 |
| Yes | 1.00 | 1.19 (0.75–1.9) | 1.29 (0.67–2.48) | 0.4117 |  | 1.00 | 1.40 (0.94–2.08) | 0.81 (0.52–1.28) | 0.2726 |  |
| **Body Mass Index** |  |  |  |  |  |  |  |  |  |  |
| < 23 kg/m^2^ | 1.00 | 0.96 (0.37–2.53) | 0.86 (0.26–2.89) | 0.8022 | 0.4388 | 1.00 | 1.81 (0.70–4.69) | 0.94 (0.35–2.55) | 0.6535 | 0.6923 |
| ≥ 23 kg/m^2^ | 1.00 | 0.98 (0.68–1.43) | 1.35 (0.78–2.34) | 0.3820 |  | 1.00 | 1.09 (0.77–1.54) | 0.77 (0.52–1.14) | 0.1723 |  |
| **Waist Circumference** | |  |  |  |  |  |  |  |  |  |
| < 90/85 cm (men/women) | 1.00 | 0.83 (0.50–1.40) | 0.81 (0.40–1.65) | 0.5362 | 0.5603 | 1.00 | 1.18 (0.71–1.95) | 0.74 (0.43–1.27) | 0.1894 | 0.7295 |
| ≥ 90/85 cm (men/women) | 1.00 | 1.18 (0.72–1.94) | 1.74 (0.88–3.46) | 0.1412 |  | 1.00 | 1.21 (0.78–1.88) | 0.87 (0.54–1.41) | 0.5338 |  |
| **Fasting Blood Glucose** | |  |  |  |  |  |  |  |  |  |
| Normoglycemia (< 100 mg/dL) | 1.00 | 1.47 (0.61–3.52) | 2.31 (0.85–6.28) | 0.0791 | 0.0527 | 1.00 | 1.02 (0.48–2.20) | 1.48 (0.72–3.01) | 0.2364 | 0.0142 |
| Prediabetes (100 to 125 mg/dL) | 1.00 | 0.90 (0.61–1.33) | 1.03 (0.55–1.92) | 0.9358 |  | 1.00 | 1.29 (0.90–1.85) | 0.61 (0.39–0.95) | 0.0255 |  |
|  |  |  |  |  |  |  |  |  |  |  |
| **CUMULATIVE AVERAGE** | |  |  |  |  |  |  |  |  |  |
| **Level of Education** |  |  |  |  |  |  |  |  |  |  |
| <12 years | 1.00 | 0.83 (0.53–1.28) | 1.42 (0.72–2.81) | 0.6794 | 0.0954 | 1.00 | 1.11 (0.71–1.75) | 1.04 (0.67–1.63) | 0.8628 | 0.6147 |
| ≥12 years | 1.00 | 1.28 (0.68–2.41) | 3.52 (1.51–8.23) | 0.0056 |  | 1.00 | 0.90 (0.53–1.53) | 1.24 (0.71–2.18) | 0.4341 |  |
| **Regular Exercise** |  |  |  |  |  |  |  |  |  |  |
| No | 1.00 | 0.90 (0.60–1.33) | 1.70 (0.97–2.97) | 0.1842 | 0.4708 | 1.00 | 0.88 (0.60–1.28) | 0.95 (0.66–1.37) | 0.8021 | 0.0643 |
| Yes | 1.00 | 1.38 (0.63–2.98) | 7.55 (2.14–26.60) | 0.0443 |  | 1.00 | 2.23 (1.04–4.78) | 3.05 (1.35–6.93) | 0.0036 |  |
| **Current Smoker** |  |  |  |  |  |  |  |  |  |  |
| No | 1.00 | 1.01 (0.65–1.58) | 2.49 (1.21–5.10) | 0.1253 | 0.8762 | 1.00 | 1.23 (0.79–1.93) | 1.46 (0.92–2.29) | 0.1018 | 0.1292 |
| Yes | 1.00 | 0.94 (0.52–1.70) | 1.74 (0.80–3.78) | 0.1911 |  | 1.00 | 0.77 (0.46–1.30) | 0.79 (0.47–1.32) | 0.4289 |  |
| **Current Drinker** |  |  |  |  |  |  |  |  |  |  |
| No | 1.00 | 0.63 (0.35–1.12) | 1.75 (0.86–3.57) | 0.4760 | 0.4884 | 1.00 | 0.57 (0.30–1.09) | 1.05 (0.60–1.84) | 0.8972 | 0.6071 |
| Yes | 1.00 | 1.24 (0.78–1.98) | 2.18 (1.06–4.49) | 0.0586 |  | 1.00 | 1.30 (0.85–1.97) | 1.20 (0.77–1.87) | 0.4287 |  |
| **Body Mass Index** |  |  |  |  |  |  |  |  |  |  |
| < 23 kg/m^2^ | 1.00 | 0.57 (0.23–1.39) | 0.84 (0.25–2.88) | 0.7974 | 0.1117 | 1.00 | 0.95 (0.37–2.43) | 0.90 (0.37–2.19) | 0.8258 | 0.2965 |
| ≥ 23 kg/m^2^ | 1.00 | 1.04 (0.71–1.52) | 2.54 (1.43–4.50) | 0.0246 |  | 1.00 | 1.03 (0.72–1.48) | 1.15 (0.79–1.67) | 0.4529 |  |
| **Waist Circumference** | |  |  |  |  |  |  |  |  |  |
| < 90/85 cm (men/women) | 1.00 | 0.62 (0.38–1.02) | 1.05 (0.50–2.20) | 0.7845 | 0.1915 | 1.00 | 0.97 (0.59–1.59) | 0.79 (0.48–1.33) | 0.3616 | 0.3943 |
| ≥ 90/85 cm (men/women) | 1.00 | 1.52 (0.91–2.53) | 4.03 (1.97–8.27) | 0.0008 |  | 1.00 | 1.15 (0.72–1.84) | 1.59 (1.00–2.52) | 0.0511 |  |
| **Fasting Blood Glucose** | |  |  |  |  |  |  |  |  |  |
| Normoglycemia (< 100 mg/dL) | 1.00 | 1.47 (0.61–3.52) | 3.32 (1.13–9.76) | 0.0276 | 0.1487 | 1.00 | 0.97 (0.44–2.13) | 1.80 (0.89–3.63) | 0.0668 | 0.0365 |
| Prediabetes (100 to 125 mg/dL) | 1.00 | 0.97 (0.66–1.42) | 1.97 (1.06–3.66) | 0.1650 |  | 1.00 | 1.08 (0.74–1.56) | 0.94 (0.63–1.40) | 0.7621 |  |
|  |  |  |  |  |  |  |  |  |  |  |
| **RECENT** |  |  |  |  |  |  |  |  |  |  |
| **Level of Education** |  |  |  |  |  |  |  |  |  |  |
| <12 years | 1.00 | 1.19 (0.79–1.78) | 1.74 (0.96–3.17) | 0.0899 | 0.2520 | 1.00 | 1.13 (0.71–1.80) | 1.50 (0.98–2.30) | 0.0609 | 0.9436 |
| ≥12 years | 1.00 | 1.38 (0.81–2.36) | 3.27 (1.60–6.69) | 0.0052 |  | 1.00 | 1.13 (0.66–1.93) | 1.49 (0.86–2.59) | 0.1534 |  |
| **Regular Exercise** |  |  |  |  |  |  |  |  |  |  |
| No | 1.00 | 1.13 (0.80–1.61) | 1.84 (1.11–3.04) | 0.0416 | 0.1664 | 1.00 | 1.14 (0.78–1.67) | 1.30 (0.90–1.89) | 0.1584 | 0.1409 |
| Yes | 1.00 | 1.99 (0.93–4.25) | 4.79(1.58–14.54) | 0.0048 |  | 1.00 | 1.10 (0.46–2.64) | 3.21 (1.53–6.75) | 0.0048 |  |
| **Current Smoker** |  |  |  |  |  |  |  |  |  |  |
| No | 1.00 | 1.36 (0.90–2.04) | 2.52 (1.37–4.66) | 0.0064 | 0.7305 | 1.00 | 0.95 (0.51–1.76) | 1.41 (0.80–2.50) | 0.2688 | 0.9244 |
| Yes | 1.00 | 1.14 (0.68–1.91) | 1.90 (0.96–3.76) | 0.1030 |  | 1.00 | 1.26 (0.82–1.93) | 1.56 (1.03–2.38) | 0.0366 |  |
| **Current Drinker** |  |  |  |  |  |  |  |  |  |  |
| No | 1.00 | 0.96 (0.55–1.66) | 1.88 (0.91–3.86) | 0.2181 | 0.5885 | 1.00 | 1.31 (0.46–3.71) | 1.98 (0.79–4.98) | 0.1333 | 0.8989 |
| Yes | 1.00 | 1.45 (0.97–2.18) | 2.30 (1.26–4.18) | 0.0055 |  | 1.00 | 1.14 (0.79–1.66) | 1.52 (1.06–2.18) | 0.0264 |  |
| **Body Mass Index** |  |  |  |  |  |  |  |  |  |  |
| < 23 kg/m^2^ | 1.00 | 1.40 (0.57–3.44) | 1.69 (0.52–5.49) | 0.3580 | 0.1812 | 1.00 | 1.31 (0.46–3.71) | 1.98 (0.79–4.98) | 0.1333 | 0.5807 |
| ≥ 23 kg/m^2^ | 1.00 | 1.28 (0.91–1.80) | 2.37 (1.44–3.91) | 0.0027 |  | 1.00 | 1.14 (0.79–1.66) | 1.52 (1.06–2.18) | 0.0264 |  |
| **Waist Circumference** | |  |  |  |  |  |  |  |  |  |
| < 90/85 cm (men/women) | 1.00 | 0.91 (0.59–1.41) | 1.21 (0.62–2.37) | 0.7793 | 0.2062 | 1.00 | 0.99 (0.60–1.62) | 1.04 (0.64–1.69) | 0.8780 | 0.4409 |
| ≥ 90/85 cm (men/women) | 1.00 | 1.93 (1.20–3.11) | 4.13 (2.22–7.69) | <.0001 |  | 1.00 | 1.42 (0.86–2.34) | 2.33 (1.45–3.74) | 0.0003 |  |
| **Fasting Blood Glucose** | |  |  |  |  |  |  |  |  |  |
| Normoglycemia (< 100mg/dL) | 1.00 | 1.49 (0.75–2.96) | 3.10 (1.29–7.41) | 0.0156 | 0.2159 | 1.00 | 1.13 (0.53–2.41) | 2.02 (1.03–3.95) | 0.0341 | 0.1078 |
| Prediabetes (100 to 125mg/dL) | 1.00 | 1.17 (0.82–1.67) | 2.04 (1.18–3.54) | 0.0289 |  | 1.00 | 1.25 (0.85–1.85) | 1.30 (0.89–1.91) | 0.1674 |  |

The multivariate-adjusted model was adjusted for age, educational level, regular exercise, smoking status, alcohol consumption, body mass index, and Diet Quality Index-International (DQI-I) score, except for the variables used in the interaction test. The stratification variable was not included as a covariate in its respective stratified model. Addiitonally, BMI and WC were not adjusted for each other in the stratified analyses

^1^ *p-*values for linear trends were obtained by treating the median value of each group as a continuous variable.

^2^ *p*-int is the p-value for the interaction.
