# Supplementary material for: Prospective associations between psychosocial stress and the risk of type 2 diabetes in middle-aged adults: findings from the KoGES_CAVAS
Source: Epidemiol Health. 2025 Oct 31;47:e2025061. doi: 10.4178/epih.e2025061 (PMC12885608; doi:10.4178/epih.e2025061)
Supplement: Supplementary Material 9. — Stratified analyses of the association between Psychosocial Well-being Index-short Form (PWI-SF) scores and type 2 diabetes risk in women [file epih-47-e2025061-Supplementary-9.docx]

**Supplementary Material 9.** Stratified analyses of the association between Psychosocial Well-being Index-short Form (PWI-SF) scores and type 2 diabetes risk in women

|  | **Categories of PWI-SF scores** | | | | | | | | | ***p*-trend^1^** | | | | ***p*-int^2^** | | | | **Tertiles of PWI-SF scores** | | | | | | | | | | ***p*-trend^1^** | | ***p*-int^2^** | |
| --- | --- | --- | --- | --- | --- | --- | --- | --- | --- | --- | --- | --- | --- | --- | --- | --- | --- | --- | --- | --- | --- | --- | --- | --- | --- | --- | --- | --- | --- | --- | --- |
|  | **Healthy group** | | **Potential stress group** | | | **High-risk group** | | | |  |  |  |  |  |  |  |  | **T1** | | **T2** | | | **T3** | | | | |  |  |  |  |
| **BASELINE** |  | |  | | |  | | | |  | | | |  | | | |  | |  | | |  | | | | |  | |  | |
| **Level of Education** |  | |  | | |  | | | |  | | | |  | | | |  | |  | | |  | | | | |  | |  | |
| <12 years | 1.00 | | 1.31 (0.82–2.07) | | | 1.35 (0.79–2.29) | | | | 0.3019 | | | | 0.7026 | | | | 1.00 | | 1.13 (0.77–1.65) | | | 1.08 (0.75–1.56) | | | | | 0.7170 | | 0.8691 | |
| ≥12 years | 1.00 | | 1.24 (0.57–2.69) | | | 1.51 (0.54–4.25) | | | | 0.4219 | | | |  |  |  |  | 1.00 | | 0.96 (0.52–1.76) | | | 1.08 (0.55–2.09) | | | | | 0.8414 | |  |  |
| **Regular Exercise** |  | |  | | |  | | | |  | | | |  | | | |  | |  | | |  | | | | |  | |  | |
| No | 1.00 | | 1.20 (0.76–1.89) | | | 1.11 (0.65–1.90) | | | | 0.8205 | | | | 0.0835 | | | | 1.00 | | 1.08 (0.75–1.57) | | | 0.98 (0.68–1.42) | | | | | 0.8467 | | 0.2495 | |
| Yes | 1.00 | | 1.47 (0.68–3.16)^4^ | | | 2.43 (0.96–6.11)^4^ | | | | 0.0568 | | | |  |  |  |  | 1.00 | | 0.99 (0.52–1.89)^4^ | | | 1.40 (0.74–2.64)^4^ | | | | | 0.3286 | |  |  |
| **Current Smoker^3^** |  | |  | | |  | | | |  | | | |  | | | |  | |  | | |  | | | | |  | |  | |
| No | 1.00 | | 1.32 (0.89–1.98) | | | 1.39 (0.87–2.24) | | | | 0.1963 | | | | - | | | | 1.00 | | 1.06 (0.77–1.47) | | | 1.07 (0.78–1.48) | | | | | 0.6662 | | - | |
| Yes | - | | - | | | - | | | | - | | | |  |  |  |  | - | | - | | | - | | | | | - | |  |  |
| **Current Drinker** |  | |  | | |  | | | |  | | | |  | | | |  | |  | | |  | | | | |  | |  | |
| No | 1.00 | | 1.38 (0.86–2.21) | | | 1.43 (0.81–2.51) | | | | 0.2503 | | | | 0.6637 | | | | 1.00 | | 1.12 (0.78–1.61) | | | 0.96 (0.65–1.41) | | | | | 0.8170 | | 0.3059 | |
| Yes | 1.00 | | 1.07 (0.52–2.19) | | | 1.23 (0.52–2.91) | | | | 0.6226 | | | |  |  |  |  | 1.00 | | 0.99 (0.50–1.95) | | | 1.44 (0.80–2.61) | | | | | 0.2119 | |  |  |
| **Body Mass Index** |  | |  | | |  | | | |  | | | |  | | | |  | |  | | |  | | | | |  | |  | |
| < 23 kg/m^2^ | 1.00 | | 0.95 (0.32–2.80)^4^ | | | 0.60 (0.13–2.83)^4^ | | | | 0.4735 | | | | 0.4487 | | | | 1.00 | | 0.69 (0.28–1.67)^4^ | | | 0.46 (0.16–1.26)^4^ | | | | | 0.1309 | | 0.5371 | |
| ≥ 23 kg/m^2^ | 1.00 | | 1.27 (0.83–1.93) | | | 1.40 (0.85–2.31) | | | | 0.1882 | | | |  |  |  |  | 1.00 | | 1.13 (0.80–1.59) | | | 1.15 (0.81–1.62) | | | | | 0.4543 | |  |  |
| **Waist Circumference** |  | |  | | |  | | | |  | | | |  | | | |  | |  | | |  | | | | |  | |  | |
| < 90/85 cm (men/women) | 1.00 | | 1.46 (0.69–3.07) | | | 1.26 (0.52–3.04) | | | | 0.7341 | | | | 0.5856 | | | | 1.00 | | 0.75 (0.44–1.30) | | | 0.91 (0.54–1.55) | | | | | 0.8118 | | 0.8827 | |
| ≥ 90/85 cm (men/women) | 1.00 | | 1.16 (0.73–1.86) | | | 1.39 (0.79–2.44) | | | | 0.2443 | | | |  |  |  |  | 1.00 | | 1.32 (0.89–1.95) | | | 1.17 (0.78–1.75) | | | | | 0.5212 | |  |  |
| **Fasting Blood Glucose** |  |  | | |  | | |  | | | |  | | | |  | | | | |  | | |  | |  | | |  |  |  |
| Normoglycemia (< 100 mg/dL) | 1.00 | | 1.92 (0.88–4.18) | | | 1.59 (0.63–4.01) | | | | 0.4426 | | | | 0.6981 | | | | 1.00 | | 1.49 (0.83–2.68) | | | 1.58 (0.87–2.87) | | | | | 0.1421 | | 0.2261 | |
| Prediabetes (100 to 125 mg/dL) | 1.00 | | 1.29 (0.82–2.03) | | | 1.60 (0.94–2.73) | | | | 0.0755 | | | |  |  |  |  | 1.00 | | 1.13 (0.77–1.67) | | | 0.99 (0.67–1.45) | | | | | 0.9607 | |  |  |
| **Menopausal Status** |  | |  | | |  | | | |  | | | |  | | | |  | |  | | |  | | | | |  | |  | |
| No | 1.00 | | 1.00 (0.45–2.25)^4^ | | | 1.18 (0.44–3.16)^4^ | | | | 0.7158 | | | | 0.8272 | | | | 1.00 | | 0.63 (0.32–1.22)^4^ | | | 0.71 (0.37–1.36)^4^ | | | | | 0.3501 | | 0.1787 | |
| Yes | 1.00 | | 1.38 (0.88–2.17) | | | 1.34 (0.78–2.30) | | | | 0.3511 | | | |  |  |  |  | 1.00 | | 1.30 (0.90–1.88) | | | 1.18 (0.80–1.72) | | | | | 0.4644 | |  |  |
|  |  | |  | | |  | | | |  | | | |  | | | |  | |  | | |  | | | | |  | |  | |
| **CUMULATIVE AVERAGE** | | | |  | | |  | | | |  | | | |  | | | |  |  | | |  | | | | |  | |  | |
| **Level of Education** |  | |  | | |  | | | |  | | | |  | | | |  | |  | | |  | | | | |  | |  | |
| <12 years | 1.00 | | 1.45 (0.87–2.43) | | | 1.92 (1.05–3.53) | | | | 0.0309 | | | | 0.3587 | | | | 1.00 | | 1.00 (0.67–1.49) | | | 1.56 (1.09–2.25) | | | | | 0.0110 | | 0.4791 | |
| ≥12 years | 1.00 | | 0.93 (0.44–1.96) | | | 0.98 (0.29–3.26) | | | | 0.9475 | | | |  |  |  |  | 1.00 | | 1.03 (0.57–1.89) | | | 1.16 (0.61–2.22) | | | | | 0.6536 | |  |  |
| **Regular Exercise** |  | |  | | |  | | | |  | | | |  | | | |  | |  | | |  | | | | |  | |  | |
| No | 1.00 | | 1.11 (0.69–1.80) | | | 1.32 (0.73–2.36) | | | | 0.3400 | | | | 0.1371 | | | | 1.00 | | 0.84 (0.57–1.23) | | | 1.31 (0.92–1.86) | | | | | 0.0967 | | 0.2274 | |
| Yes | 1.00 | | 1.79 (0.76–4.22)^4^ | | | 3.23 (1.06–9.83)^4^ | | | | 0.0279 | | | |  |  |  |  | 1.00 | | 1.62 (0.86–3.05)^4^ | | | 2.00 (1.03–3.90)^4^ | | | | | 0.0343 | |  |  |
| **Current Smoker^3^** |  | |  | | |  | | | |  | | | |  | | | |  | |  | | |  | | | | |  | |  | |
| No | 1.00 | | 1.31 (0.86–2.02) | | | 1.67 (0.98–2.84) | | | | 0.0561 | | | | - | | | | 1.00 | | 1.00 (0.71–1.40) | | | 1.46 (1.06–2.01) | | | | | 0.0164 | | - | |
| Yes | - | | - | | | - | | | | - | | | |  |  |  |  | - | | - | | | - | | | | | - | |  |  |
| **Current Drinker** |  | |  | | |  | | | |  | | | |  | | | |  | |  | | |  | | | | |  | |  | |
| No | 1.00 | | 1.37 (0.83–2.26) | | | 1.66 (0.89–3.10) | | | | 0.1059 | | | | 0.7481 | | | | 1.00 | | 1.14 (0.77–1.68) | | | 1.58 (1.08–2.30) | | | | | 0.0149 | | 0.4990 | |
| Yes | 1.00 | | 1.02 (0.46–2.28) | | | 1.43 (0.53–3.82) | | | | 0.4521 | | | |  |  |  |  | 1.00 | | 0.76 (0.40–1.43) | | | 1.23 (0.70–2.16) | | | | | 0.4409 | |  |  |
| **Body Mass Index** |  | |  | | |  | | | |  | | | |  | | | |  | |  | | |  | | | | |  | |  | |
| < 23 kg/m^2^ | 1.00 | | 0.58 (0.20–1.63)^4^ | | | 0.70 (0.16–3.16)^4^ | | | | 0.6809 | | | | 0.8814 | | | | 1.00 | | 0.38 (0.13–1.11)^4^ | | | 1.07 (0.45–2.53)^4^ | | | | | 0.8525 | | 0.7655 | |
| ≥ 23 kg/m^2^ | 1.00 | | 1.37 (0.86–2.19) | | | 1.67 (0.94–2.97) | | | | 0.0778 | | | |  |  |  |  | 1.00 | | 1.11 (0.78–1.59) | | | 1.46 (1.04–2.06) | | | | | 0.0248 | |  |  |
| **Waist Circumference** |  | |  | | |  | | | |  | | | |  | | | |  | |  | | |  | | | | |  | |  | |
| < 90/85 cm (men/women) | 1.00 | | 1.08 (0.53–2.20) | | | 1.12 (0.44–2.84) | | | | 0.8172 | | | | 0.8682 | | | | 1.00 | | 0.63 (0.36–1.12) | | | 1.06 (0.63–1.77) | | | | | 0.7215 | | 0.9917 | |
| ≥ 90/85 cm (men/women) | 1.00 | | 1.28 (0.76–2.15) | | | 1.73 (0.91–3.29) | | | | 0.0813 | | | |  |  |  |  | 1.00 | | 1.33 (0.88–2.03) | | | 1.75 (1.16–2.63) | | | | | 0.0063 | |  |  |
| **Fasting Blood Glucose** | | | |  | | |  | |  | | | |  | | | |  | | | | |  | | |  | |  | | | |  |
| Normoglycemia (< 100 mg/dL) | 1.00 | | 1.49 (0.68–3.30) | | | 2.04 (0.79–5.29) | | | | 0.1302 | | | | 0.9916 | | | | 1.00 | | 1.34 (0.73–2.47) | | | 2.04 (1.13–3.67) | | | | | 0.0133 | | 0.3054 | |
| Prediabetes (100 to 125 mg/dL) | 1.00 | | 1.38 (0.85–2.25) | | | 1.95 (1.04–3.66) | | | | 0.0334 | | | |  |  |  |  | 1.00 | | 1.11 (0.74–1.65) | | | 1.36 (0.93–1.97) | | | | | 0.1064 | |  |  |
| **Menopausal status** |  | |  | | |  | | | |  | | | |  | | | |  | |  | | |  | | | | |  | |  | |
| No | 1.00 | | 0.58 (0.28–1.22)^4^ | | | 1.28 (0.50–3.27)^4^ | | | | 0.6032 | | | | 0.5265 | | | | 1.00 | | 0.89 (0.46–1.70)^4^ | | | 0.91 (0.48–1.74)^4^ | | | | | 0.7912 | | 0.1055 | |
| Yes | 1.00 | | 1.67 (1.00–2.79) | | | 1.72 (0.90–3.26) | | | | 0.1106 | | | |  |  |  |  | 1.00 | | 1.05 (0.71–1.55) | | | 1.64 (1.13–2.37) | | | | | 0.0060 | |  |  |
|  |  | |  | | |  | | | |  | | | |  | | | |  | |  | | |  | | | | |  | |  | |
| **RECENT** |  | |  | | |  | | | |  | | | |  |  |  |  |  | |  | | |  | | | | |  | |  |  |
| **Level of Education** |  | |  | | |  | | | |  | | | |  | | | |  | |  | | |  | | | | |  | |  | |
| <12 years | 1.00 | | 1.61 (1.10–2.35) | | | 1.94 (1.19–3.17) | | | | 0.0037 | | | | 0.3660 | | | | 1.00 | | 1.46 (1.00–2.14) | | | 1.90 (1.32–2.72) | | | | | 0.0004 | | 0.3918 | |
| ≥12 years | 1.00 | | 1.01 (0.54–1.88) | | | 1.17 (0.44–3.14) | | | | 0.7868 | | | |  |  |  |  | 1.00 | | 1.01 (0.55–1.88) | | | 1.28 (0.68–2.42) | | | | | 0.4521 | |  |  |
| **Regular Exercise** |  | |  | | |  | | | |  | | | |  | | | |  | |  | | |  | | | | |  | |  | |
| No | 1.00 | | 1.44 (0.99–2.11) | | | 1.53 (0.93–2.53) | | | | 0.0611 | | | | 0.3082 | | | | 1.00 | | 1.28 (0.88–1.88) | | | 1.74 (1.21–2.49) | | | | | 0.0024 | | 0.9970 | |
| Yes | 1.00 | | 1.39 (0.72–2.66)^4^ | | | 2.77 (1.16–6.60)^4^ | | | | 0.0301 | | | |  |  |  |  | 1.00 | | 1.48 (0.79–2.78)^4^ | | | 1.73 (0.91–3.28)^4^ | | | | | 0.0829 | |  |  |
| **Current Smoker^3^** |  | |  | | |  | | | |  | | | |  | | | |  | |  | | |  | | | | |  | |  | |
| No | 1.00 | | 1.45 (1.05–2.02) | | | 1.73 (1.12–2.68) | | | | 0.0073 | | | | - | | | | 1.00 | | 1.33 (0.95–1.84) | | | 1.74 (1.27–2.38) | | | | | 0.0005 | | - | |
| Yes | - | | - | | | - | | | | - | | | |  |  |  |  | - | | - | | | - | | | | | - | |  |  |
| **Current Drinker** |  | |  | | |  | | | |  | | | |  | | | |  | |  | | |  | | | | |  | |  | |
| No | 1.00 | | 1.5 (1.02–2.20) | | | 1.92 (1.17–3.16) | | | | 0.0062 | | | | 0.3406 | | | | 1.00 | | 1.45 (0.99–2.11) | | | 1.81 (1.25–2.62) | | | | | 0.0014 | | 0.6958 | |
| Yes | 1.00 | | 1.23 (0.65–2.32) | | | 1.11 (0.44–2.79) | | | | 0.7130 | | | |  |  |  |  | 1.00 | | 1.03 (0.55–1.95) | | | 1.50 (0.83–2.74) | | | | | 0.1767 | |  |  |
| **Body Mass Index** |  | |  | | |  | | | |  | | | |  | | | |  | |  | | |  | | | | |  | |  | |
| < 23 kg/m^2^ | 1.00 | | 0.89 (0.36–2.20)^4^ | | | 1.85 (0.57–5.96)^4^ | | | | 0.4381 | | | | 0.9288 | | | | 1.00 | | 0.64 (0.23–1.78)^4^ | | | 1.61 (0.68–3.76)^4^ | | | | | 0.2818 | | 0.6388 | |
| ≥ 23 kg/m^2^ | 1.00 | | 1.44 (1.02–2.05) | | | 1.64 (1.03–2.61) | | | | 0.0223 | | | |  |  |  |  | 1.00 | | 1.41 (1.00–1.99) | | | 1.67 (1.19–2.34) | | | | | 0.0026 | |  |  |
| **Waist Circumference** |  | |  | | |  | | | |  | | | |  | | | |  | |  | | |  | | | | |  | |  | |
| < 90/85 cm (men/women) | 1.00 | | 1.08 (0.64–1.82) | | | 1.07 (0.49–2.33) | | | | 0.8314 | | | | 0.5285 | | | | 1.00 | | 0.94 (0.56–1.61) | | | 1.12 (0.66–1.89) | | | | | 0.6854 | | 0.9233 | |
| ≥ 90/85 cm (men/women) | 1.00 | | 1.54 (1.01–2.36) | | | 2.11 (1.24–3.60) | | | | 0.0039 | | | |  |  |  |  | 1.00 | | 1.53 (1.01–2.31) | | | 2.12 (1.42–3.15) | | | | | 0.0002 | |  |  |
| **Fasting Blood Glucose** | | | |  | | |  | | | |  | | | |  | | | |  |  | | |  | | | | |  | |  | |
| Normoglycemia (< 100 mg/dL) | 1.00 | | 1.53 (0.86–2.73) | | | 1.74 (0.81–3.74) | | | | 0.1142 | | | | 0.6972 | | | | 1.00 | | 1.53 (0.85–2.73) | | | 2.13 (1.22–3.72) | | | | | 0.0066 | | 0.5110 | |
| Prediabetes (100 to 125 mg/dL) | 1.00 | | 1.47 (1.00–2.17) | | | 1.99 (1.19–3.34) | | | | 0.0060 | | | |  |  |  |  | 1.00 | | 1.36 (0.91–2.03) | | | 1.62 (1.11–2.36) | | | | | 0.0111 | |  |  |
| **Menopausal status** |  | |  | | |  | | | |  | | | |  | | | |  | |  | | |  | | | | |  | |  | |
| No | 1.00 | | 1.04 (0.53–2.03)^4^ | | | 1.32 (0.52–3.34)^4^ | | | | 0.5915 | | | | 0.4721 | | | | 1.00 | | 0.84 (0.43–1.66)^4^ | | | 1.25 (0.66–2.39)^4^ | | | | | 0.4859 | | 0.3262 | |
| Yes | 1.00 | | 1.56 (1.07–2.26) | | | 1.80 (1.10–2.95) | | | | 0.0091 | | | |  |  |  |  | 1.00 | | 1.50 (1.04–2.18) | | | 1.87 (1.30–2.68) | | | | | 0.0006 | |  |  |

The multivariate-adjusted model was adjusted for age, educational level, regular exercise, smoking status, alcohol consumption, body mass index, and Diet Quality Index-International (DQI-I) score, except for the variables used in the interaction test. The stratification variable was not included as a covariate in its respective stratified model. Addiitonally, BMI and WC were not adjusted for each other in the stratified analyses.

^1^ *p-*values for linear trends were obtained by treating the median value of each group as a continuous variable.

^2^ *p*-int is the p-value for the interaction.

^3^ The association and interactions were not analyzed because of the lack of current smokers and incident cases among women.

^4^ Marked analyses were conducted using the multivariable-adjusted model; however, smoking status was excluded because of statistical errors (insufficient number of current smokers and incident cases among women).
