# Supplementary material for: Prospective associations between psychosocial stress and the risk of type 2 diabetes in middle-aged adults: findings from the KoGES_CAVAS
Source: Epidemiol Health. 2025 Oct 31;47:e2025061. doi: 10.4178/epih.e2025061 (PMC12885608; doi:10.4178/epih.e2025061)
Supplement: Supplementary Material 10. [file epih-47-e2025061-Supplementary-10.docx]

**Supplementary Material 10-1.** The dose-response association of psychosocial stress (PWI-SF score) with incident type 2 diabetes in overall men [(A) ~ (C)] and women [(D) ~ (F)] using restricted cubic spline analysis with three knots (located at the 25^th^, 50^th^ and 75^th^ percentiles).


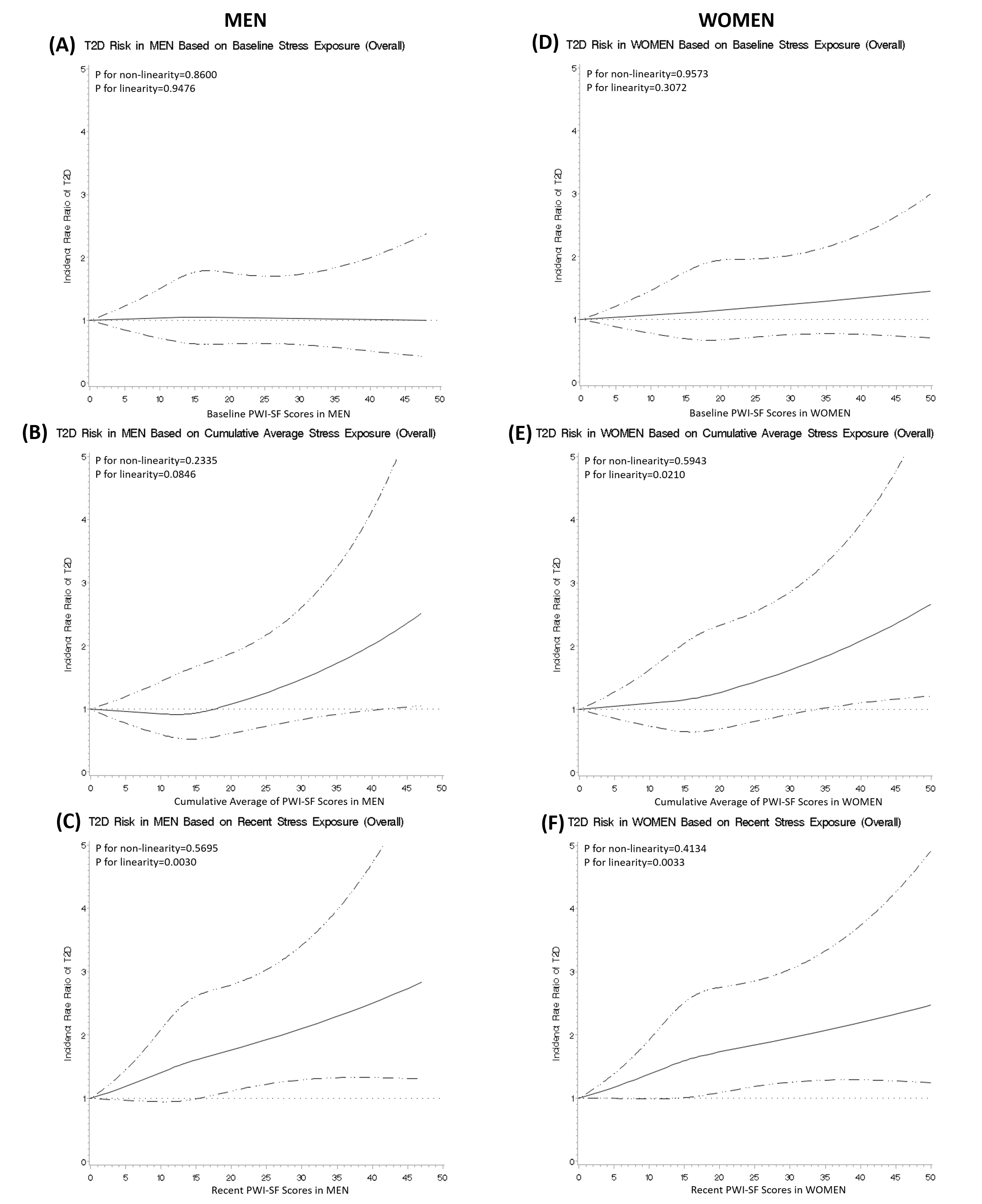


Multivariable models were adjusted for age, education (≥12 years), regular exercise (≥3 times/week, ≥30 min/session), smoking (current, past, or never for men; yes or no for women), alcohol intake (mL/day), body mass index (kg/m²), and Diet Quality Index- International (DQI-I).

**
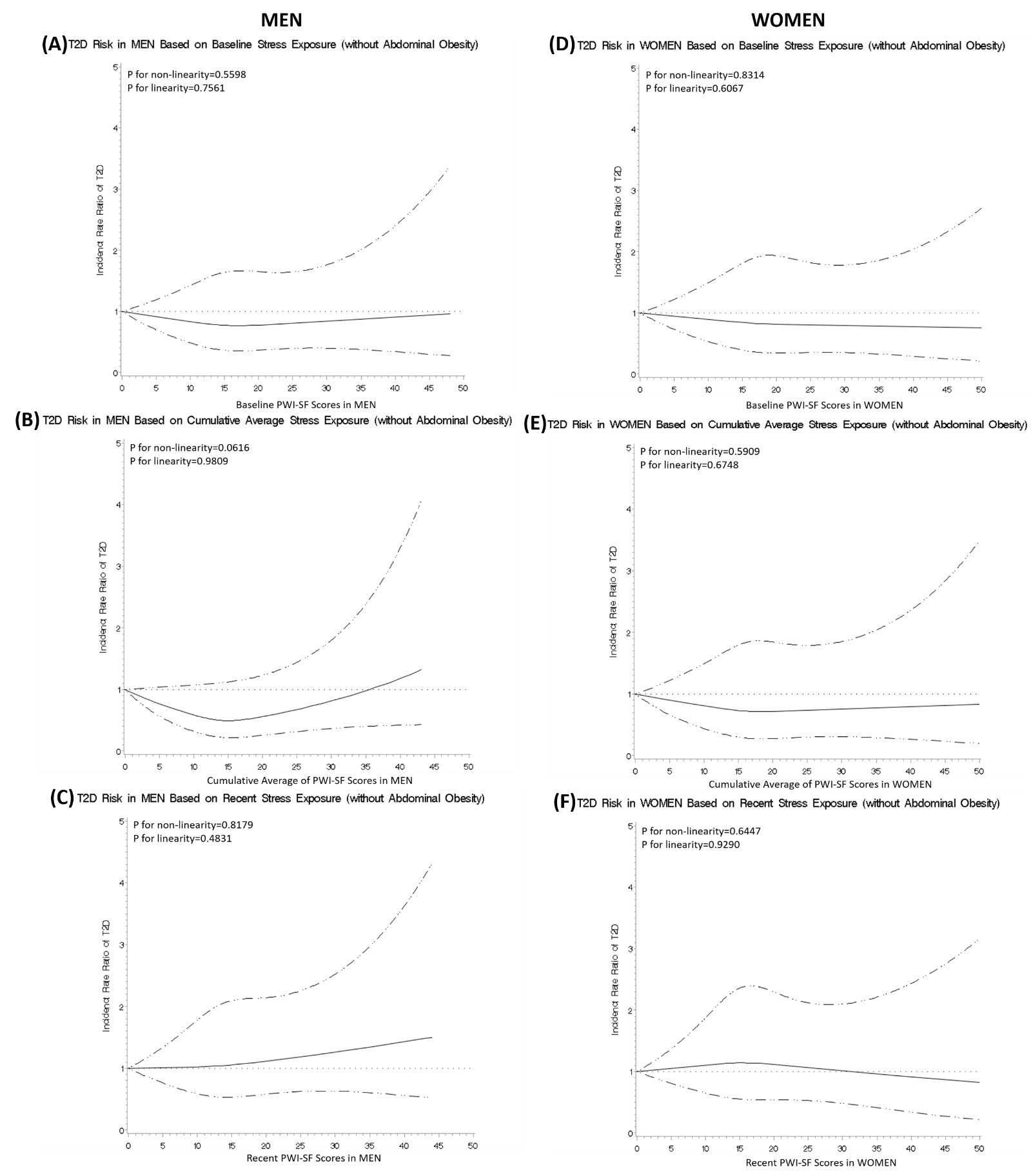
Supplementary Material 10-2.** The dose-response association of psychosocial stress (PWI-SF score) with incident type 2 diabetes in normal (without abdominal obesity) men [(A) ~ (C)] and women [(D) ~ (F)] using restricted cubic spline analysis with three knots (located at the 25^th^, 50^th^ and 75^th^ percentiles).

Multivariable models were adjusted for age, education (≥12 years), regular exercise (≥3 times/week, ≥30 min/session), smoking (current, past, or never for men; yes or no for women), alcohol intake (mL/day), body mass index (kg/m²), and Diet Quality Index- International (DQI-I).

**
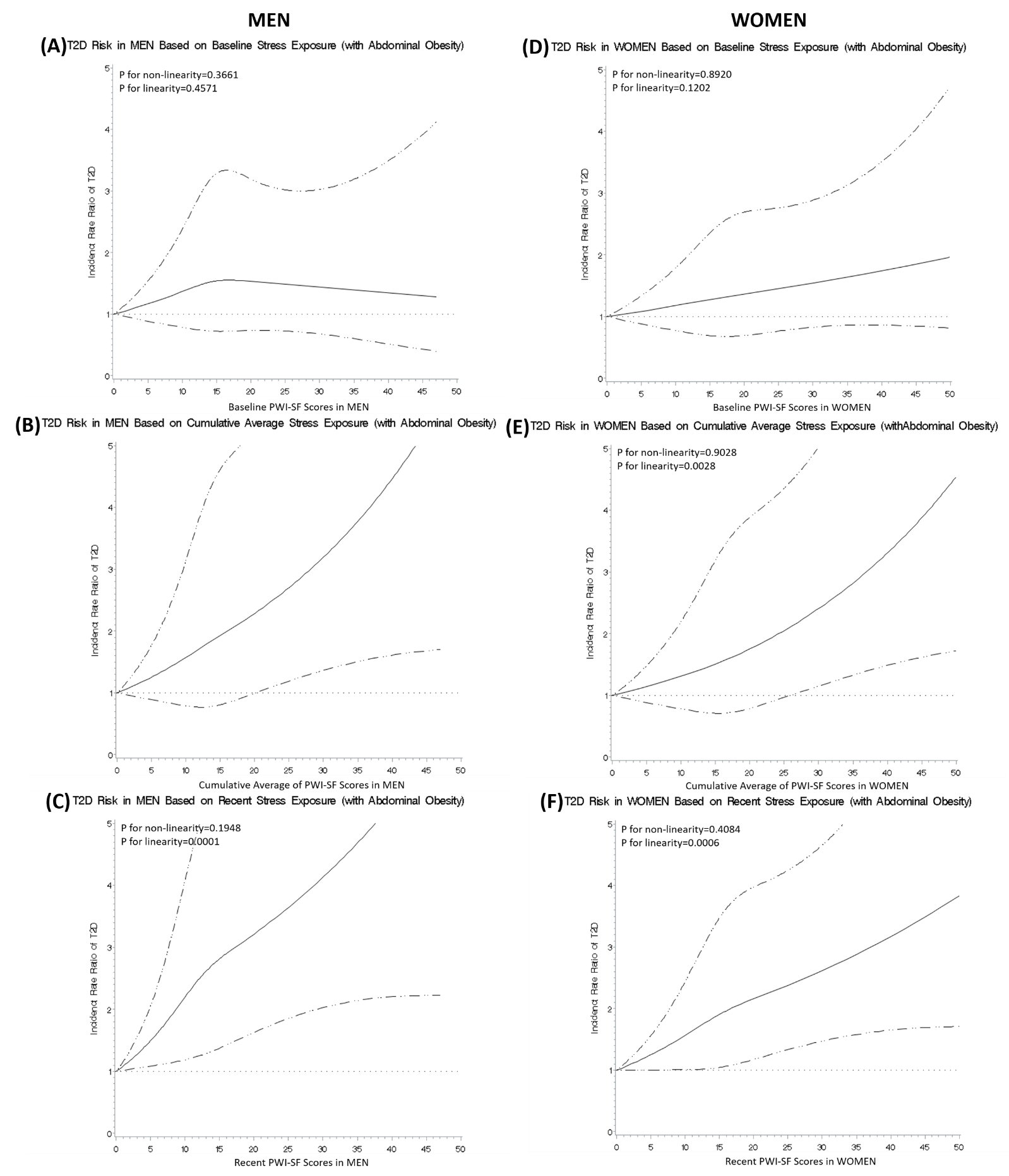
Supplementary Material 10-3.** The dose-response association of psychosocial stress (PWI-SF score) with incident type 2 diabetes in obese (with abdominal obesity) men [(A) ~ (C)] and women [(D) ~ (F)] using restricted cubic spline analysis with three knots (located at the 25^th^, 50^th^ and 75^th^ percentiles).

Multivariable models were adjusted for age, education (≥12 years), regular exercise (≥3 times/week, ≥30 min/session), smoking (current, past, or never for men; yes or no for women), alcohol intake (mL/day), body mass index (kg/m²), and Diet Quality Index- International (DQI-I)
