# Supplementary material for: Prospective associations between psychosocial stress and the risk of type 2 diabetes in middle-aged adults: findings from the KoGES_CAVAS
Source: Epidemiol Health. 2025 Oct 31;47:e2025061. doi: 10.4178/epih.e2025061 (PMC12885608; doi:10.4178/epih.e2025061)
Supplement: Supplementary Material 11. — Incidence rate ratios of type 2 diabetes by psychosocial stress levels (baseline, cumulative average, and recent PWI-SF scores) from multiple imputation analysis [file epih-47-e2025061-Supplementary-11.docx]

**Supplementary Material** **11.** Incidence rate ratios of type 2 diabetes by psychosocial stress levels (baseline, cumulative average, and recent PWI-SF scores) from multiple imputation analysis

|  | **Categories of PWI-SF scores** | | | **Tertiles of PWI-SF scores** | | |
| --- | --- | --- | --- | --- | --- | --- |
|  | **Healthy group** | **Potential stress group** | **High-risk group** | **T1** | **T2** | **T3** |
|  | **(0.0≤PWI-SF≤8.0)** | **(8.0<PWI-SF<27.0)** | **(27.0≤PWI-SF≤54.0)** |  |  |  |
| **MEN** |  |  |  |  |  |  |
| Baseline | 1.00 | 0.95 (0.67–1.34) | 1.11 (0.67–1.84) | 1.00 | 1.07 (0.78–1.48) | 0.82 (0.57–1.17) |
| Cumulative Average | 1.00 | 0.99 (0.69–1.43) | 2.01 (1.17–3.46) | 1.00 | 1.02 (0.73–1.43) | 1.14 (0.82–1.60) |
| Recent | 1.00 | 1.26 (0.92–1.73) | 2.26 (1.45–3.53) | 1.00 | 1.15 (0.82–1.64) | 1.50 (1.08–2.08) |
|  |  |  |  |  |  |  |
| **WOMEN** |  |  |  |  |  |  |
| Baseline | 1.00 | 1.23 (0.84–1.81) | 1.41 (0.89–2.22) | 1.00 | 1.04 (0.75–1.44) | 1.07 (0.78–1.46) |
| Cumulative Average | 1.00 | 1.31 (0.82–2.09) | 1.69 (1.00–2.86) | 1.00 | 1.10 (0.82–1.47) | 1.37 (1.00–1.88) |
| Recent | 1.00 | 1.39 (0.97–1.98) | 1.77 (1.14–2.76) | 1.00 | 1.27 (0.94–1.72) | 1.67 (1.22–2.27) |

Multivariable model: adjusted for age, education level, regular exercise, smoking status, alcohol consumption, body mass index (BMI) and Diet Quality Index – International (DQI-I)
