# Supplementary material for: Prospective associations between psychosocial stress and the risk of type 2 diabetes in middle-aged adults: findings from the KoGES_CAVAS
Source: Epidemiol Health. 2025 Oct 31;47:e2025061. doi: 10.4178/epih.e2025061 (PMC12885608; doi:10.4178/epih.e2025061)
Supplement: Supplementary Material 12. — Methods for multiple imputation [file epih-47-e2025061-Supplementary-12.docx]

**Supplementary Material 12.** Methods for multiple imputation

*Multiple imputation*

Multiple imputation was performed using a fully conditional specification (FCS) approach, stratified by sex. Binary categorical variables such as education level, regular exercise, and smoking status for women were imputed using logistic regression, while the multinomial categorical variable such as smoking status for men (current, former, never) was imputed using a discriminant distribution. A total of five imputed datasets (nimpute = 5, seed = 12345) were generated. Each imputation model included the outcome (incident T2D), exposures (baseline, cumulative average, and recent PWI-SF scores), covariates (age, higher education, regular exercise, smoking status, alcohol consumption, BMI, and DQI-I), and log-transformed person-years as an offset term. For variables with non-negative constraints (e.g., alcohol consumption, PWI-SF scores), lower bounds were set to zero. Modified Poisson regression with a log link and robust error estimator was applied to each dataset, and incidence rate ratios (IRRs) with 95% confidence intervals were pooled across imputations using Rubin’s rules.
